# Disability reduction following a lumbar stabilization exercise program for low back pain: large vs. small improvement subgroup analyses of physical and psychological variables

**DOI:** 10.1186/s12891-024-07480-4

**Published:** 2024-05-04

**Authors:** Christian Larivière, Richard Preuss, Marie-France Coutu, Michael J. Sullivan, Nicolas Roy, Sharon M. Henry

**Affiliations:** 1 Institut de recherche Robert-Sauvé en santé et en sécurité du travail (IRSST), 505, boul. De Maisonneuve Ouest, Montreal, QC H3A 3C2 Canada; 2 School of Physical & Occupational Therapy, McGill University, 845 Sherbrooke Wst, Montreal, QC H3G 1Y5 Canada; 3 Charles-Le Moyne Hospital Research Centre, University of Sherbrooke, 150 Place Charles-Le Moyne, Office 200, Longueuil, QC J4K 0A8 Canada; 4 Department of Psychology, McGill University, 1205 Docteur Penfield, Montreal, QC H3A 1B1 Canada; 5 Department of Neurological Sciences, University of Vermont, Burlington, VT 05401 USA; 6 Center for Interdisciplinary Research in Rehabilitation of Greater Montreal (CRIR), 6363, Hudson Road, office 061, Montreal, QC H3S 1M9 Canada

**Keywords:** Lumbar stability exercise program, Treatment response, Subgroup analyses, Psychological measures, Physical tests

## Abstract

**Background:**

Little is known about why patients with low back pain (LBP) respond differently to treatment, and more specifically, to a lumbar stabilization exercise program. As a first step toward answering this question, the present study evaluates how subgroups of patients who demonstrate large and small clinical improvements differ in terms of physical and psychological changes during treatment.

**Methods:**

Participants (*n* = 110) performed the exercise program (clinical sessions and home exercises) over eight weeks, with 100 retained at six-month follow-up. Physical measures (lumbar segmental instability, motor control impairments, range of motion, trunk muscle endurance and physical performance tests) were collected twice (baseline, end of treatment), while psychological measures (fear-avoidance beliefs, pain catastrophizing, psychological distress, illness perceptions, outcome expectations) were collected at four time points (baseline, mid-treatment, end of treatment, follow-up). The participants were divided into three subgroups (large, moderate and small clinical improvements) based on the change of perceived disability scores. ANOVA for repeated measure compared well-contrasted subgroups (large vs. small improvement) at different times to test for SUBGROUP × TIME interactions.

**Results:**

Statistically significant interactions were observed for several physical and psychological measures. In all these interactions, the large- and small-improvement subgroups were equivalent at baseline, but the large-improvement subgroup showed more improvements over time compared to the small-improvement subgroup. For psychological measures only (fear-avoidance beliefs, pain catastrophizing, illness perceptions), between-group differences reached moderate to strong effect sizes, at the end of treatment and follow-up.

**Conclusions:**

The large-improvement subgroup showed more improvement than the small-improvement subgroup with regard to physical factors typically targeted by this specific exercise program as well as for psychological factors that are known to influence clinical outcomes.

**Supplementary Information:**

The online version contains supplementary material available at 10.1186/s12891-024-07480-4.

## Introduction

Low back pain (LBP) remains one of the most common and incapacitating health conditions worldwide [1]. There is strong evidence to recommend exercise programs after the acute phase, at least for reducing pain and disability [2]. However, the treatment effects for any exercise program are moderate at best for non-acute and non-specific LBP [3] and initial reviews/meta-analyses have not demonstrated the superiority of one type of exercise program over another [3, 4]. Consequently, to increase the positive impact of any treatment for LBP, research needs to determine who is likely to benefit from these treatments (e.g., specific, matched exercise interventions) based on clinical presentation and to identify the mechanisms of benefit responsible for clinical improvement.

One active exercise modality, lumbar stabilization exercise programs (LSEPs), has a solid scientific foundation and has been in use for several years [5–7]. Interestingly, although the quality of the evidence is low [8], two meta-analyses support the effectiveness of the two components that make up most LSEPs, namely coordination/stabilization exercises and strength/resistance exercises [8, 9]. Effectively, LSEPs target motor control and coordination of the paraspinal and abdominal muscles and may also progressively overload muscles to enhance trunk muscle endurance [5–7].

As the most recent Cochrane systematic review on the topic points out [10], an evidence-based patient/intervention linkage is still not established for identifying patients who respond best to a LSEP. A preliminary clinical prediction rule (CPR), at the derivation stage (*n* = 54 participants), has been proposed [11], but its formal validation was unsuccessful due to a lack of statistical power [12]. Our group has more recently conducted a cohort (observational) study to derive CPRs of success (or large improvement) at the end of the eight-week treatment (*n* = 110 participants) and at the six-month follow-up (*n* = 100 participants), using disability as the main clinical outcome [13]. Several candidate predictors of clinical success were measured at baseline for the CPRs development but were also measured at mid-treatment (psychological variables) as well at the end of treatment (physical and psychological variables) and six-month follow-up (psychological variables). Some of these measures may explain clinical improvement for such a specific exercise program, such as decreased aberrant movements or pain during movements, increased trunk muscle endurance or decreased fears, psychological distress and illness perceptions, to name a few. The design of this study does not allow identification of the corresponding mechanisms of benefit as this would require a randomized controlled trial (RCT) [14]. However, as a preliminary step toward identifying these mechanisms, the present study aims at exploring how subgroups of patients showing large and small clinical improvements in terms of disability also differ in terms of physical and psychological changes during treatment.

## Materials and methods

Most of the study methodology is summarized here. The reader is referred to Larivière, Rabhi [13] for more details.

### Design of the study

**Fig. 1:**
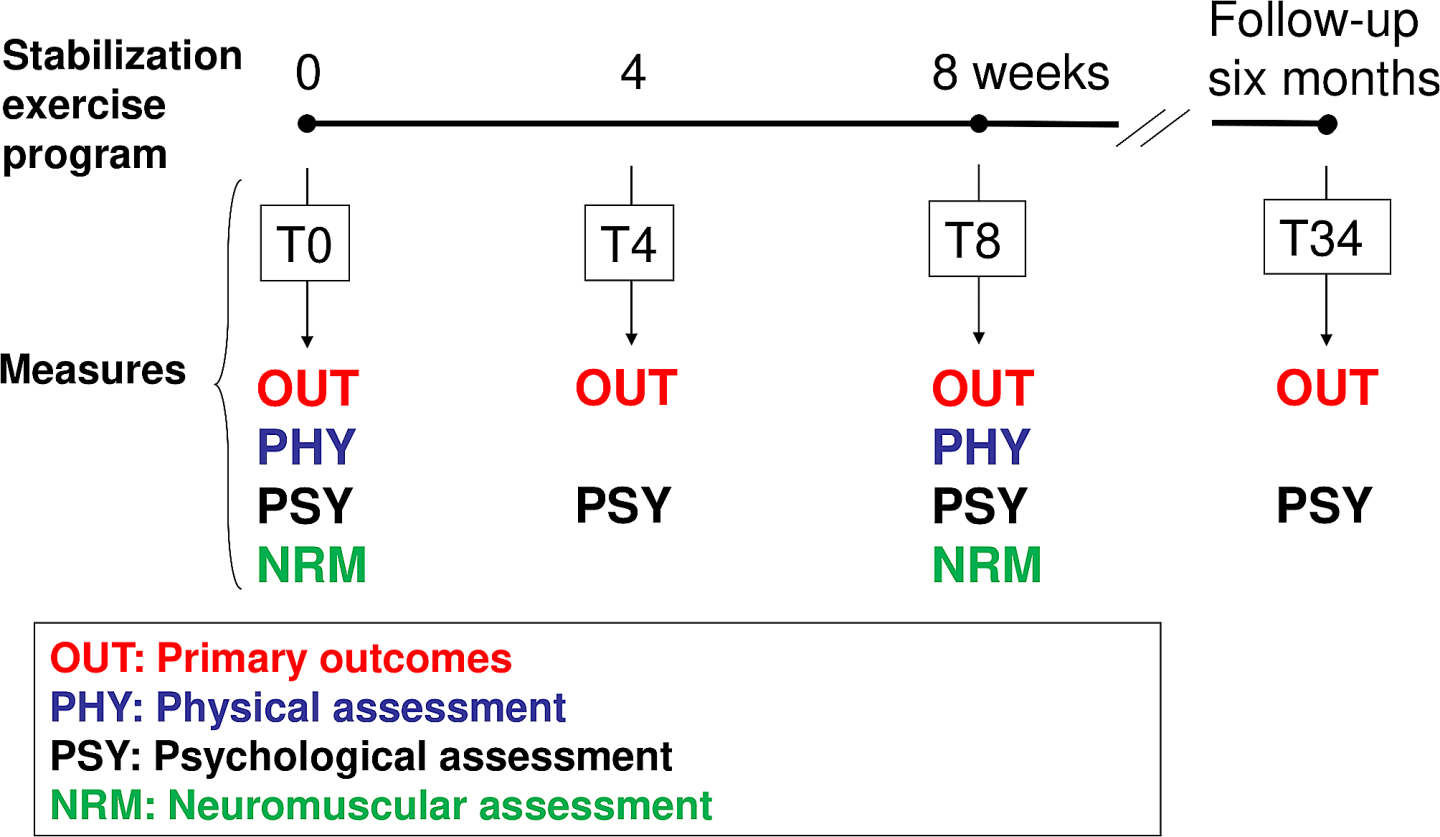
From Larivière, Rabhi [13]. Four categories of measures (OUT, PHY, PSY, NRM) were collected at different times during (T0, T4, T8) and following (T34) the lumbar stabilization exercise program. PHY and NRM measures are more burdensome and were consequently only collected at T0 and T8. NRM measures were not considered in the present study

A per-protocol prospective cohort/observational study was carried out with three assessments during the intervention (T0, T4 and T8 weeks) and one assessment at six-month follow-up (T34) (Fig. 1).

### Participants

Participants were recruited and assessed from July 2012 to August 2016 (preliminary study or phase 1) and from July 2018 to October 2020 (phase 2). They were French- or English-speaking and aged between 18 and 65 years. They had lumbar or lumbosacral pain for at least four weeks (non-acute phase), with or without radicular pain. They had a minimum score of 12% on the Oswestry Disability Index (ODI) [15] to allow a minimal clinically important change of 10% [16] to occur. Exclusion criteria were: a specific lumbar pathology (fracture, infection or tumor) or scoliosis; surgery on the pelvis or spinal column; systemic or degenerative disease; thoracic or neck pain that is more severe than LBP; pregnancy; starting an exercise program within the last six months; litigation relative to the back injury. A final exclusion criterion was the presence of one positive neurological sign in two of three test categories: (a) reduced Achilles and patellar tendon reflexes, (b) reduced strength in lumbosacral myotomes, (c) reduced sensation in lumbosacral dermatomes.

### Lumbar stabilization exercise program (LSEP)

An eight-week individualized LSEP (two, 30-min sessions/week) was provided in local physiotherapy clinics. No co-intervention was allowed except medication. The exercise program has been thoroughly described elsewhere in a supplementary file linked to a previous publication ( [17]; see Additional file 1). Briefly, The LSEP comprised three distinct phases: (1) Pain Management. over a maximum period of two weeks: This involved isolated contractions of the transversus abdominis (TrA) and lumbar multifidus (LuM) muscles followed by gradual incorporation of TrA/LuM co-contractions during low-load exercises.; (2) Early Impairment and Functional Level: Exercises emphasized quality of movement control; (3) Moderate/Advanced Impairment and Functional Level: As patients progressed, the program shifted to muscle endurance. Exercises were designed with higher volume and intensity, all while maintaining co-contractions of TrA and LuM. All the participants reached the third (final) phase during the 8-week program. The participants were also encouraged to do the exercises at home.

In order to standardize the information given to participants on their condition, the Back Book booklet [18], or its French translation (*Guide du dos* : ISBN: 978-2-923465-03-6), was given to participants at their first clinical visit. The booklet aimed to change beliefs and behaviours (resuming activities) related to back pain.

### Home-exercise adherence

Home-exercise adherence was assessed twice: once after the exercise program (T8), to measure adherence during the 8-week program, and once at six-month follow-up (T34), to measure adherence following the 8-week program. Questions about home-exercise adherence were not asked during the 8-week treatment so as not to influence participant behavior [19]. The question was “How many times have you done your exercises as prescribed in the last week?”. The physiotherapists’ prescription was based on each participant’s needs. Exercises were generally prescribed every day during phase I (pain management and motor control of deep muscles), and three times a week during phases II (initiation of exercises with emphasis on quality of movement control) and III (endurance development with emphasis on quantity). Based on the most common definition of adherence, i.e. “the extent to which the patient follows medical instructions” [20], the frequency per week was divided by the physiotherapist’s recommendation to obtain a ratio. The ratio may vary between 0 and 1, 1 being given when the frequency was equal to or higher than prescribed. Every participant reached phase III, wherein the prescribed frequency was generally three times a week. Consider this scenario: If the recommended exercise frequency was three sessions per week (as typically observed during week eight of the clinical program), and the patient actually completed only 2 sessions per week, the resulting adherence ratio would be 0.66 (equivalent to 2 out of 3 sessions).

### Assessments

A detailed description of the questionnaires used to measure the clinical outcomes (OUT) and psychological (PSY) variables and of the tests used during the physical examination (PHY) is provided in the **Supplementary file**. These variables are summarized in Table 1.

**Table 1:** Summary of outcome (OUT) measures, psychosocial (PSY) constructs, physical (PHY) tests and their acronyms (details in the **Supplementary file)**

Variable	Questionnaire (PSY measure) or test (PHY measure)	Acronym (class A, B, C)
**Clinical outcome (OUT) measures**		
Perceived disability	Oswestry Disability Index [21]	ODI (n.a.)
Pain intensity (NPRS)	Numeric Pain Rating Scale (NPRS) [22]	NPRS (n.a.)
Activity-related pain	Activity-Related Pain questionnaire [23]	ActRelPain (n.a.)
Risk of unfavorable prognosis	Subgroups for Targeted Treatment (STarT) Back Screening Tool [24]	STarT Back (n.a.)
**Psychological (PSY) measures**		
Fear and avoidance of physical activity	Fear-Avoidance Beliefs Questionnaire – physical activity subscale [25]	FABQ-PA (B)
Pain catastrophizing	Pain Catastrophizing Scale [26]	PCS (B)
Psychological distress	Psychological Distress Inventory (subscales cognitive, anxiety, depression, anger, somatization and total score) [27]	PDIcog, PDIanx, PDIdep, PDIang, PDIsoma, PDItot (B)
Illness perception	Brief-Illness Perception Questionnaire (total score) [28]	B-IPQ (B)
Habitual physical activity during sport and leisure activities	Habitual physical activity questionnaire [29]; sport and leisure activities	HPA-sport, HPA -leisure (B)
Outcome expectations related to the exercise program	Outcome Expectations for Exercise Scale [30]	Treatment expectation (B)
**Physical (PHY) measures - The different tests were performed in the following sequence:**		
Posterior chain mobility	Passive straight-leg raise [31] – ROM at pain onset	PSLR-Pain ROM-min (C)
Posterior chain mobility	Passive straight-leg raise [31] – maximal ROM tolerated after pain onset	PSLR-Max ROM-min (C)
Load transfer of lumbo-pelvic area	Active straight-leg raise [32] – ability on a 6-point scale	ASLR-Act/5-max (A)
Load transfer of lumbo-pelvic area	Active straight-leg raise [32] – pain or not	ASLR-Act-Pain-max (A)
Posture	Lumbar lordosis [33]	Lordosis (A)
Trunk range of motion	Pelvis and lumbar flexion ROM [34]	PelvisFlx-ROM, LumbFlx-ROM (C)
Trunk range of motion	Lumbar lateral flexion ROM [35]:	LumbLatFlx-ROM-Min (C)
Lumbar instability	Prone instability test [36]	ProneIT (A)
Lumbar instability	Passive lumbar extension [37]	PLE (A)
Motor control impairments	Passive and active knee flexion [38]	MCI-KneeF-Pas-Max, …Act-Max (A)
Motor control impairments	Passive and active hip internal rotation [38]	MCIP-HipIR-Pas-max, …Act-max (A)
Motor control impairments	Passive and active hip external rotation [38]	MCIP-HipER-Pas-max, …Act-max (A)
Hip range of motion	Passive hip internal rotation	MCIP-HipIR-Pas-ROM-min (C)
Hip range of motion	Passive hip external rotation	MCIP-HipER-Pas-ROM-min (C)
Motor control impairments	Passive and active hip extension [38]	MCIP-HipE-Pas-max, …Act-max (A)
Motor control impairments	Quadruped kneeling and active arm lifting [39]	MCI4-ShoF-Act-max (A)
Motor control impairments	Passive and active knee extension [40]	MCIT-KneeE-Pas-max, …Act-max (A)
Motor control impairments	Passive and active hip abduction + rotation [38]	MCIS-HipAR-Pas-max, …Act-max (A)
Ligamentous laxity	Beighton scale [41]	Beighton (A)
Aberrant movements	Aberrant movements [41]	Abe-Mvt (A)
Physical performance test	Repeated sit-to-stand [42]	PPT- SitStand (A)
Physical performance test	Repeated trunk flexion [42]	PPT-Flexions (A)
Physical performance test	Loaded reach [42]	PPT-Reach (A)
Physical performance test	360° rollover [42]	PPT-Rollover-max (A)
Trunk muscle endurance	Side bridge [43]	TME-Side-min (A)
Trunk muscle endurance	Trunk flexors [43]	TME-Abdominals (A)
Trunk muscle endurance	Back extensors [44]:	TME-Back (A)

A detailed description of the questionnaires used to measure the clinical outcomes (OUT) and psychosocial (PSY) constructs and of the tests used during the physical examination (PHY) is provided in the **Supplementary file**. **Act** : active; **ASLR**: active straight leg rising; **HipAR**: hip abduction + rotation; **HipER**: hip external rotation; **HipE**: hip extension; **HipIR**: hip internal rotation; **KneeE**: knee extension; **KneeF**: knee flexion; **L/R** : left and right; **LSI**: lumbar segmental instability; **MCI**: motor control impairment; **MCI4**: motor control impairment in quadruped kneeling baseline position; **MCIP**: motor control impairment in prone baseline position; **MCIS**: motor control impairment in supine baseline position; **MCIT**: motor control impairment in sitting baseline position; **TME**: trunk muscle endurance; **Pas**: passive; **PLE**: Passive lumbar extension; **PPT**: physical performance test; **PSLR**: passive straight leg rising; **ROM**: range of motion; **ShoF**: shoulder flexion

To reduce the likelihood of spurious findings at the derivation stage of the CPRs, the potential variables were selected according to a sound theoretical rationale using well-known theoretical models, namely the neuromuscular spine instability model [45, 46], the fear-avoidance model of pain [47] and the common-sense model of illness [48, 49], the latter being used to predict treatment adherence [50]. They were classified as Class-A, B and C variables, depending on their relationship with the theoretical background (or potential mechanisms of benefit) of the LSEP. Class A were physical variables specifically (theoretically) associated with the treatment, either in direct or indirect relation to lumbar stability. Class B are psychological variables potentially related to adherence and, as such, may influence outcomes through adherence to the home exercise program [51, 52]. Class C were the other physical variables, namely the range of motion at different joints here, that were not theoretically associated with this treatment and, as such, may be associated with other exercise programs. This A-B-C classification can be used as our current study hypotheses, namely that the large-improvement group (LIG) would show more improvement in Class-A and Class-B variables than the small-improvement group (SIG), while this would not be case for Class-C variables.

### Clinical outcome (OUT) measures

The ODI [15], a self-report measure of disability, was used to define subgroups. Pain intensity was measured for the last week preceding each time point, using an 11-point (0 to 10) numeric pain rating scale (NPRS). To relate pain to activities, participants were also asked whether they had experienced increased pain (yes/no) during general activity or exercise. Finally, to get an aggregate measure of physical and psychological factors, the Subgroups for Targeted Treatment (STarT) Back Screening Tool [24] was used to assess the risk of unfavorable prognosis.

### Physical assessment (PHY measures) for Class-A and Class-C variables

The physical examination comprised tests that can be theoretically related to lumbar segmental instability (LSI) or motor control impairments (MCI) [53–55], all with acceptable interrater reliability [kappa > 0.6; intraclass correlation coefficients - ICC > 0.70; [53]], as detailed in the **Supplementary file**. PHY testing covered different dimensions as follows: (1) LSI (*n* = 4) (2), MCI (*n* = 7) (3), posture and range of motion (ROM) (*n* = 6) (4), trunk muscle endurance (TME) (*n* = 4) and (5) physical performance tests (PPT) (*n* = 4). Regarding MCI tests, only symptoms caused by these tests were considered because the assessment of clinical signs (alignment, movements) is less reliable [56]. MCI tests are named “motor control impairment” because they are used within a clinical system whose overall goal is to determine whether the individual can actively control the kinematic chain in a manner that generally favours movement in the extremities and stability in the spine. Measures taken from both sides of the body (e.g., right and left lateral trunk flexion; left and right lower extremity measurements) were managed with the goal of retaining the measurements most associated with impairments. Specifically, only the minimal ROM (exception: lateral trunk flexion) and TME scores across left and right sides, as well as the maximal scores during PPT (related to slow movements) and MCI tests were selected for further analyses.

### Psychological assessment (PSY measures) for Class-B variables

Patient-reported outcome measures included variables from the fear-avoidance model (pain catastrophizing, fear-avoidance beliefs of physical activity, psychological distress, habitual physical activity) and variables theoretically related to home-exercise adherence [57–60]: illness perception and outcome expectations related to the exercise program.

### Statistics

The sample size was determined to have enough statistical power for the derivation of the CPRs, as detailed elsewhere [13], using preliminary findings [21] derived from 64 participants distributed in four subgroups (see next paragraph), namely large improvement (*n* = 31), moderate improvement (*n* = 5), small improvement (*n* = 12), and dropouts (*n* = 16). The intent was to have enough participants in the ‘large’ and ‘small’ subgroups to derive the CPRs. These numbers were also considered suitable for the purpose of conducting subgroup analyses based on ANOVAs for repeated measures.

At two time points (T8 or T34), three subgroups of participants were defined according to their level of improvement using ODI relative to baseline (T0): (1) large-improvement group (LIG) (2), moderate-improvement group, and (3) small-improvement group (SIG). For each participant, the ODI change score (e.g., ODI = ODIT0 - ODIT8) and the corresponding percentage [eg, ODI% = ((ODIT0 - ODIT8) / ODIT0) × 100] were calculated. A 50% improvement threshold with the ODI has been used previously [11] and more recently substantiated as a valid criterion for defining clinical success in participants with LBP [61]. Also considered was a clinically important change of 10 points in ODI scores [16]. These criteria were used to define the three subgroups:

- Large-Improvement Group (LIG): ODI% ≥ 50.
- Moderate-Improvement Group: ODI% < 50%, but ODI ≥ 10.
- Small-Improvement Group (SIG): ODI% < 50% and ODI < 10.

For continuous PHY and PSY measures, two-way ANOVAs for repeated measures on the TIME factor were modulated based on available data at the four time points as follows: for PHY measures, 2 SUBGROUP (SIG: *n* = 45; LIG: *n* = 54) × 2 TIME (T0 and T8) ANOVAs; for PSY measures, 2 SUBGROUP × 4 TIME (T0, T4, T8 and T34).

For categorical PHY and PSY variables, a mixed statistical model is not available; thus, only the TIME factor was studied, for each subgroup (SIG and LIG) separately. Although many tests produce dichotomous scores (0 = negative test; 1 = positive test), MCI test scores have three levels (-1: pain decreases; 0: same pain; 1: pain increases). These scores were first dichotomized to separate participants with a positive test (score of 1) from other participants (0 assigned to scores of 0 and − 1). The McNemar test was then applied to the PHY variables (time T0 versus T8) while the Cochran Q test was applied to the PSY variables (T0, T4, T8 and T34) in order to compare the proportion of positive tests between the measurement times.

Because there was no control group composed of participants who did not receive the treatment, it was not possible to attribute the TIME effect to time or to treatment, which is why “time/treatment effect” is used in the following text.

For a clearer interpretation of the magnitude of the effects (for continuous variables), effect sizes were calculated using formulations analogous to Cohen’s *d*, namely the Hedges’ g_s_ (for independent groups) and g_av_ (for repeated measures), allowing for comparisons between within-subjects and between-subjects effects [62]. Like Cohen’s *d*, a g value of 0.2–0.5 is interpreted as a “low” effect, 0.5–0.80 “average” and > 0.8 “strong” [62]. To facilitate interpretation, g values to describe the TIME effect were calculated so that negative values indicate a decrease over time.

## Results

### Participants characteristics, home-exercise adherence and effect of the LSEP on clinical outcomes

The participants meeting the criteria for LIG, moderate improvement and SIG at T8 were 54 (23 males + 31 females), 11 (4 M + 7 F) and 45 (23 M + 22 F), respectively. At T34, they were 53 (25 M + 28 F), 11 (1 M + 10 F) and 36 (16 M + 20 F), respectively.

Demographic, anthropometric and clinical characteristics of the participants are described in Table 2, which demonstrates that all characteristics were equivalent between the LIG and SIG. The sex distribution was also equivalent between the LIG (23 M, 31 F) and SIG (23 M, 22 F), as tested with the Wald Chi-square test (*P* = 0.397). For the duration of the self-reported LBP, 98% (108/110) of participants had chronic pain (3 months or more), distributed as follows [63]: less than one month (*n* = 0), 1–3 months (*n* = 2), 3–6 months (*n* = 2), 6–12 months (*n* = 12), 1–5 years (*n* = 40),  > 5 years (*n* = 54).

**Table 2:** Demographic, anthropometric and clinical characteristics of the participants

Variables	SIG (*n* = 45)			LIG (*n* = 54)		T-test
	Mean	(SD)		Mean	(SD)	*P* value
Age (yrs)	43	(12)		43	(12)	0.961
Height (m)	1.69	(0.08)		1.69	(0.09)	0.789
Mass (kg)	74	(13)		78	(17)	0.217
BMI (kg/m^2^) *	25.7	(4.0)		26.8	(5.0)	0.235
ODI (%)	23.7	(8.7)		27.4	(9.8)	0.050
NPRS (score /10)	5.3	(1.4)		4.8	(1.2)	0.114
StarTBack (score/9)	4.2	(2.0)		3.8	(2.0)	0.397

* SIG and LIG: small- and large-improvement groups; BMI: Body mass index; ODI: Oswestry disability index; NPRS: Numerical pain rating score

As it was a per-protocol observational study, all the 110 participants attended at least 14 out of the 16 physiotherapy treatments over the eight-week LSEP. One-hundred participants reached the six-month follow-up. The ratio of home-exercise adherence did not differ between subgroups at either T8 (SIG: 0.79 ± 0.31; LIG: 0.82 ± 0.27; *P* = 0.604) or T34 (SIG: 0.43 ± 0.40; LIG: 0.55 ± 0.39; *P* = 0.165).

For all participants as well as for the three subgroups (large, moderate and small improvement), a detailed description of the effect of the LSEP on ODI and NPRS outcome measures is provided elsewhere [13]. For the study population as a whole, the LSEP generated strong clinical effects. The ODI decreased significantly (*P* < 0.05) from baseline to T8 (*n* = 110; Cohen’s *d* = -1.24) and from baseline to T34 (*n* = 100; *d* = -1.24). Significant improvements were also observed for NPRS, with corresponding *d* scores showing strong effect sizes (-1.70 at T8 and − 1.32 at T34).

### Clinical outcome (OUT) measures

#### Continuous variables

**Fig. 2:**
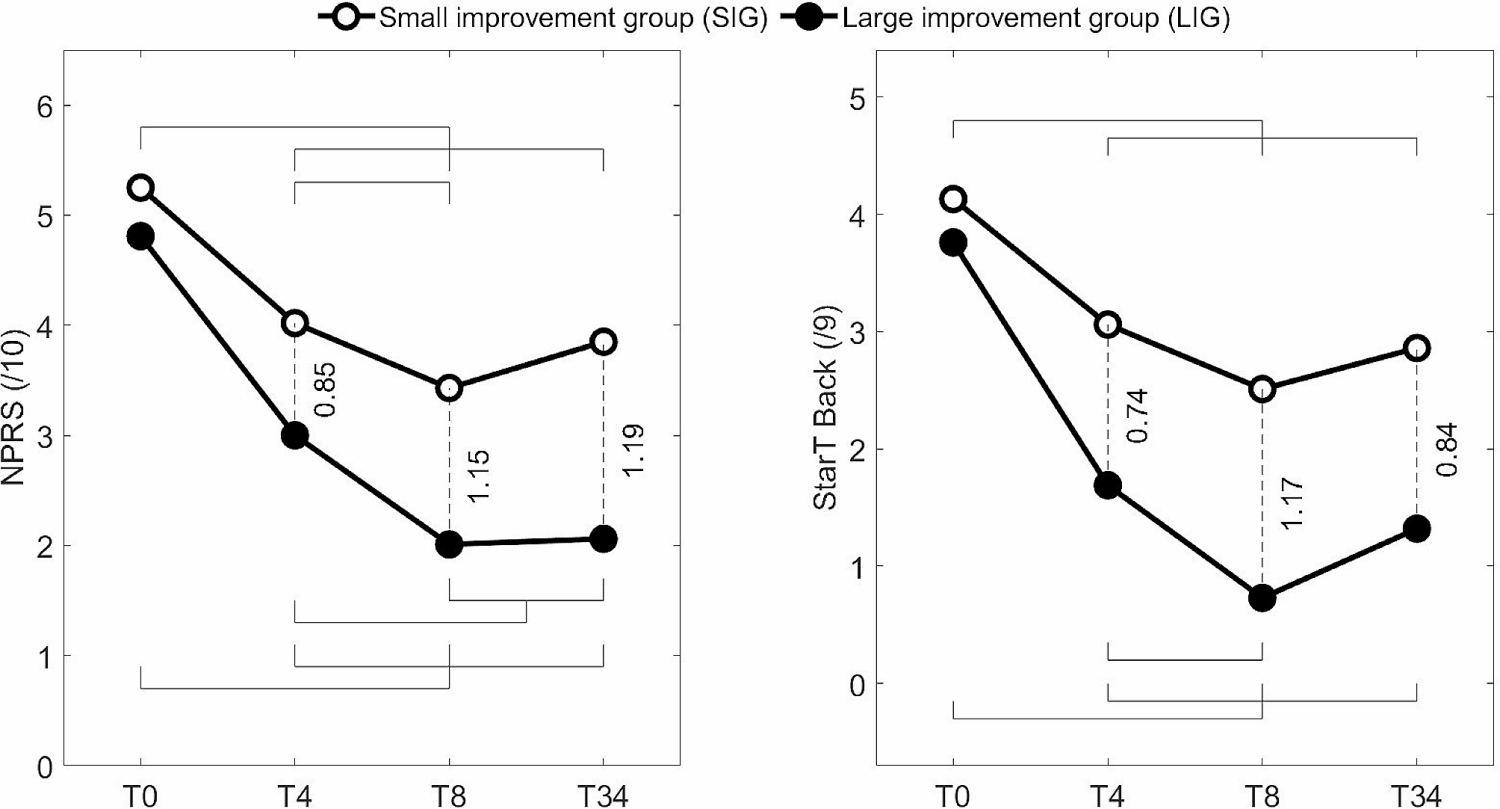
Statistically significant SUBGROUP × TIME interactions obtained for the Numeric pain rating scale (NPRS) and prognostic screening tool (STarT Back). Standard deviations were not shown for clarity. Significant differences as detected with post hoc tests are identified with the corresponding g values (effect sizes) for between-subgroup differences and by horizontal lines for between-time differences. For example, the horizontal lines on the upper left plot indicate significantly higher pain intensity at T0 comparatively to T4, T8 and T34 as well as higher pain at T4 than at T8

Results corresponding to the ODI are not considered here as the ODI was used to determine the LIG and SIG. A statistically significant SUBGROUP (SIG, LIG) × TIME (T0, T8) interaction was observed for pain intensity (NPRS) and the STarT Back screening tool, as illustrated in Fig. 2. For both measures, the LIG showed progressively lower scores than the SIG as time elapsed, reaching moderate to strong effect size.

#### Dichotomous variables

The proportion of LIG participants having pain during physical activity (ActRelPain) decreased over time (77, 56, 25, and 23% for times T0, T4, T8, and T34, respectively), which appears to be better than the decrease obtained in the SIG (70, 59, 48, and 41%), but no nonparametric test allows for this two-factor (SUBGROUP × TIME) comparison. Although each of the comparisons (Cochran’s test) led to a significant TIME main effect (P < 0.001), post hoc tests (McNemar), combined with an adjustment of alpha for the number of comparisons (*n* = 6, so *P* = 0. 05/6 = 0.00833), were significant only for the LIG (T0 = T4; T0 < T8 and T34; T0 < T8 and T34; T8 = T34).

### Physical assessment (PHY measures)

#### Continuous variables

**Fig. 3:**
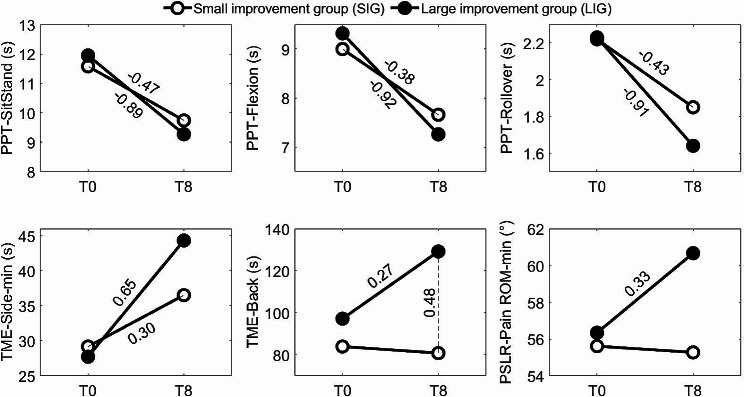
SUBGROUP × TIME interactions obtained for the continuous PHY variables. Standard deviations were not shown for clarity. Significant differences obtained with post hoc tests are identified with the corresponding g values (effect sizes). TME: trunk muscle endurance; PPT: physical performance test

The SUBGROUP (SIG, LIG) × TIME (T0, T8) ANOVAs revealed six SUBGROUP × TIME interactions (Table 3), as shown in Fig. 3. In all cases, the SIG and LIG were not different at T0, but all measures showed a more favorable effect for the LIG than for the SIG.

The effect of time/treatment, without interaction with the SUBGROUP factor, was also statistically significant for six other variables (Table 3), all indicating improvement over time. Although the corresponding effect sizes were small for most of them, i.e., for TME-Abdominals (g = 0.15; from T0: 56 ± 55 s to T8: 65 ± 68 s), PSLR-Pain ROM-min (g = 0.18 from T0: 72 ± 15° to T8: 74 ± 14°), PelvisFlx-ROM (g = 0.35; from T0: 77 ± 17° to T8 : 83 ± 15°), LumbLatFlx-ROM-Min (g = 0.31; from T0: 23 ± 9° to T8: 25 ± 10°), and MCIP-HipER-Pas-ROM-min (g = 0.27; from T0: 59 ± 10° to T8: 62 ± 10°), it was average for ASLR-Act/5-max (g = -0.53; from T0: 0.91 ± 1.20 /5 to T8: 0.39 ± 0.76 /5).

#### Dichotomous variables

For the vast majority of these tests, the LIG showed a significant improvement from T0 to T8, whereas the SIG showed no effect (Table 4).

### Psychological assessment (PSY measures)

**Fig. 4:**
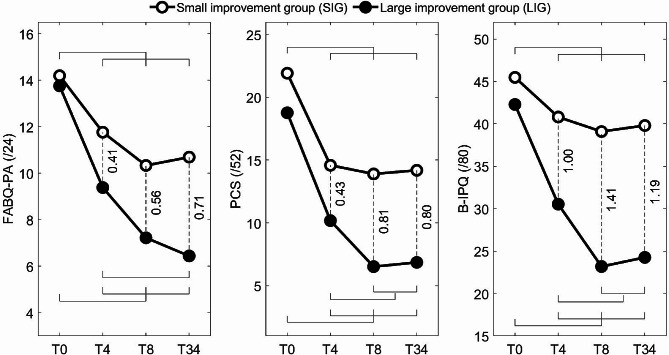
Statistically significant SUBGROUP × TIME interactions obtained for continuous PSY variables. Standard deviations were not shown for clarity. Significant differences as detected with post hoc tests are identified with the corresponding g values (effect sizes) for between-subgroup differences and by horizontal lines for between-time differences. For example, the horizontal lines on the lower left plot indicate significantly higher FABQ-PA scores at T0 comparatively to T4, T8 and T34 as well as higher scores at T4 than at T34. FABQ-AP: fears-avoidance beliefs about physical activity; PCS: pain catastrophizing scale; B-IPQ: Brief illness perception questionnaire

The SUBGROUP (SIG, LIG) × TIME (T0, T4, T8, T34) ANOVAs revealed three statistically significant interactions (Table 5; Fig. 4). They included fear-avoidance beliefs about physical activity (FABQ-AP), pain catastrophizing (PCS) and illness perceptions (B-IPQ). These interactions all behave similarly as LIG and SIG were equivalent at T0, but the LIG showed more improvements over time compared to the SIG, reflected in the differences between groups, detected as early as T4, increased until T8 and finally stabilized at T34 (Fig. 4).

**Table 3:** Effect of subgroup and time/treatment on PHY measures (continuous variables) collected at times T0 and T8

Variables	ANOVA*P* values		
	SUBGROUP (SubG)	TIME (T)	SubG × T
	(g effect size †)	(g effect size ‡)	
**Class-A variables**			
Beighton (score/75)	*P* = 0.370 (g = -0.05)	*P* = 0.890 (g = 0.02)	*P* = 0.320
Lordosis (°)	*P* = 0.305 (g = 0.18)	*P* = 0.404 (g = -0.08)	*P* = 0.496
PPT-Reach (cm)	*P* = 0.501 (g = -0.17)	*P* = 0.367 (g = 0.01)	*P* = 0.211
PPT- SitStand (s)	*P* = 0.801 (g = 0.01)	*P* < **0.001** (g = -0.67)	*P* = **0.002** ‼
PPT-Flexions (s)	*P* = 0.512 (g = 0.01)	*P* < **0.001** (g = -0.60)	*P* = **0.009** ‼
PPT-Rollover-max (s)	*P* = 0.474 (g = 0.13)	*P* < **0.001** (g = -0.65)	*P* = **0.024** ‼
TME- Side-min (s)	*P* = 0.595 (g = -0.12)	*P* < **0.001** (g = 0.50)	*P* = **0.048** ‼
TME-Abdominals (s)	*P* = 0.335 (g = -0.29)	*P* < **0.001** (g = 0.15)	*P* = 0.161
TME-Back (s)	*P* = 0.291 (g = -0.31)	*P* < **0.001** (g = 0.16)	*P* = **0.010** ‼
ASLR-Act/5-max	*P* = 0.221 (g = 0.27)	*P* < **0.001** (g = -0.53)	*P* = 0.766
**Class-C variables**			
PSLR-Pain ROM-min (°)	*P* = 0.216 (g = -0.24)	*P* = 0.056 (g = 0.17)	*P* = **0.043** ‼
PSLR-Max ROM-min (°)	*P* = 0.623 (g = -0.08)	*P* = **0.016** (g = 0.18)	*P* = 0.060
PelvisFlx-ROM (°)	*P* = 0.872 (g = -0.01)	*P* < **0.001** (g = 0.35)	*P* = 0.117
LumbFlx-ROM (°)	*P* = 0.907 (g = 0.01)	*P* = 0.436 (g = -0.06)	*P* = 0.643
LumbLatFlx-ROM-Min (°)	*P* = 0.485 (g = 0.09)	*P* < **0.001** (g = 0.31)	*P* = 0.415
MCIP-HipIR-Pas-ROM-min (°)	*P* = 0.223 (g = 0.26)	*P* = 0.576 (g = 0.05)	*P* = 0.545
MCIP-HipER-Pas-ROM-min (°)	*P* = 0.105 (g = 0.27)	*P* = **0.001** (g = 0.27)	*P* = 0.721

g effect size of 0.2–0.5 is interpreted as a “low” effect, 0.5–0.80 “average” and ˃ 0.8 “strong”

† Positive value when SIG > LIG and negative when SIG < LIG

‡ Negative values indicate a decrease over time

‼ Interaction shown in Fig. 2

*P* values ≤ 0.05 are in bold type

**Table 4:** Effect of time/treatment on PHY measures (dichotomous Class-A variables) collected at times T0 and T8

Variables*	Proportion (%) of positive tests								
	Small-improvement group (SIG)					Large-improvement group (LIG)			
	T0	T8	T8-T0†	*P* value (McNemar)		T0	T8	T8-T0†	*P* value (McNemar)
ProneIT	57	57	0	0.796		37	46	10	0.353
PLE	48	36	-11	0.166		44	21	-23	**0.016**
Abe-Mvt	18	18	0	0.739		40	13	-27	**0.005**
ASLR-Act-Pain-max	23	14	-9	0.405		48	12	-37	**< 0.001**
MCI-KneeF-Pas-Max	34	23	-11	0.157		31	13	-17	**0.013**
MCI-KneeF-Act-Max	34	23	-11	0.132		46	8	-38	**< 0.001**
MCIP-HipIR-Pas-max	57	34	-23	**0.004**		54	23	-31	**0.003**
MCIP-HipIR-Act-max	34	16	-18	**0.021**		37	12	-25	**0.007**
MCIP-HipER-Pas-max	30	25	-5	0.593		40	8	-33	**< 0.001**
MCIP-HipER-Act-max	34	30	-5	0.593		48	12	-37	**< 0.001**
MCIP-HipE-Pas-max	41	45	5	0.683		35	8	-27	**0.001**
MCIP-HipE-Act-max	82	50	-32	**< 0.001**		75	23	-52	**< 0.001**
MCI4-ShoF-Act-max	20	2	-18	**0.011**		35	8	-27	**0.005**
MCIT-KneeE-Pas-max	34	25	-9	0.248		52	23	-29	**0.001**
MCIT-KneeE-Act-max	34	20	-14	0.083		50	17	-33	**0.002**
MCIS-HipAR-Pas-max	20	18	-2	0.782		50	10	-40	**< 0.001**
MCIS-HipAR-Act-max	20	20	0	1.000		40	12	-29	**0.009**

* *P* values ≤ 0.05 are in bold type

† Change in proportion (%) of participants with a positive test between times T0 and T8; therefore, a negative value indicates improvement

**Table 5:** Effect of subgroup (SubG) and time/treatment (T) on continuous Class-B PSY measures collected at times T0, T4, T8 and T34

Variables	ANOVA *P* values			Post-hoc tests †	g effect size ‡
	SUBGROUP (SubG) (g effect size*)	TIME (T) T0 vs. T4 vs. T8 vs. T34	SubG × T	(TIME effect)	(TIME) T8 - T0
FABQ-AP (/24)	*P* = **0.002** (g = 0.41)	*P* < **0.001**	*P* = **0.021**‼	T4. T8. T34	g = -0.93
PCS (/52)	*P* < **0.001** (g = 0.51)	*P* < **0.001**	*P* = **0.001**‼	T4. T8. T34	g = -0.95
PDIcog (/100)	*P* = **0.041** (g = 0.20)	*P* = **0.032**	*P* = 0.186	/	g = − 0.26
PDIanx (/100)	*P* = 0.085 (g = 0.16)	*P* < **0.001**	*P* = 0.435	T4. T8. T34	g = -0.37
PDIdep (/100)	*P* = **0.005** (g = 0.30)	*P* = **0.011**	*P* = 0.377	T8	g = -0.18
PDIang (/100)	*P* < **0.001** (g = 0.50)	*P* = **0.010**	*P* = 0.131	T8	g = -0.21
PDIsoma (/100)	*P* = **0.037** (g = 0.25)	*P* < **0.001**	*P* = 0.248	T4. T8. T34	g = -0.55
PDItot (/100)	*P* = **0.022** (g = 0.33)	*P* < **0.001**	*P* = 0.073	T4. T8. T34	g = -0.41
B-IPQ (/80)	*P* < **0.001** (g = 0.91)	*P* < **0.001**	*P* < **0.001**‼	T4. T8. T34	g = -1.16
HPA-sport (/5)	*P* = 0.560 (g = -0.12)	*P* = 0.274	*P* = 0.050	/	g = 0.01
HPA-leisure (/5)	*P* = 0.855 (g = 0.02)	*P* = 0.394	*P* = 0.138	/	g = − 0.05
Treatment expectations (1–5)	*P* = 0.928 (g = -0.05)	*P* = **0.033**	*P* = 0.634	/	g = 0.23

g effect size of 0.2–0.5 is interpreted as a “low” effect, 0.5–0.80 “average” and > 0.8 “strong”

* Positive value when SIG > LIG and negative when SIG < LIG

† Only significant differences from T0 are identified

‡ Negative values indicate a decrease over time

‼ Interaction shown in Fig. 3

*P* values ≤ 0.05 are in bold type

Other SUBGROUP effects were observed (Table 5), but without interacting with the TIME factor, for almost all the variables related to psychological distress (PDIcog, PDIdep, PDIang, PDIsoma, PDItot). Given the absence of significant interaction, these variables were more favorable in the LIG from the start and for all measurement times, but the effect sizes were small (g between 0.20 and 0.50).

The effect of time/treatment, without interaction with the SUBGROUP factor, was also statistically significant for a majority of the continuous PSY measures investigated (Table 5), indicating improvement over time. These included all variables related to psychological distress (PDItot and scores on the five subscales) and treatment expectations, although post-hoc analyses did not reach statistical significance for the latter. Effect sizes at T8 (relative to T0) were strong (g between − 0.93 and − 1.16) for FABQ-AP, PCS and B-IPQ.

## Discussion

The main findings of the present study indicate that participants in the LIG showed greater improvements than those in the SIG, both with regard to the physical factors targeted by this specific exercise program and in some psychological factors known to potentially influence clinical outcomes. Taken together, these findings support our hypotheses and suggest that both physical and psychological variables may have influenced the improvement in back pain related disability (ODI).

Ideally, mediation analyses are conducted to confirm the presence of mechanisms of benefit of a given treatment, provided that a randomized controlled trial (RCT) design is used so the intervention-outcome and intervention mediator effects can be assumed to be unconfounded [14]. As concluded in a recent review on this topic [64], very few RCTs have evaluated the mechanisms of benefit underlying different exercise programs in participants with chronic LBP. Although the present study is not an RCT, it does allow, through the comparison of large-improvement (LIG) and small-improvement (SIG) subgroups, to test specific hypotheses related to the variables that may contribute to clinical improvement during a LSEP. This study design did not include randomization, so it cannot be ascertained if all potential confounders were equivalent between our LIG and SIG. However, it should be recalled that this study adopted a per-protocol approach, which is good practice for CPR development. In other words, participants who failed to attend clinical appointments were rejected, representing seven cases here [13]. Even home-exercise adherence was equivalent between groups at T8 and T34, rejecting adherence as a potential mediating factor. Also, these groups were equivalent at baseline for general characteristics (demographic, anthropometric), clinical outcome measures (ODI, NPRS, StarTBack) as well as for all variables (physical and psychological) tested. Of particular note, the groups were equivalent at baseline for the StartBack screening tool, an aggregate measure of the most recognized prognostic factors for LBP disability.

Although the subgroups were determined on the basis of self-reported disability, a greater decrease in pain symptoms was also observed in the LIG than in the SIG during the LSEP and up to the six-month follow-up. Consistent with these results, the proportion of LIG participants having pain during physical activity (ActRelPain) significantly decreased over time, contrary to the SIG. Therefore, although the targeted physical and psychological variables were related to self-reported disability in the present study, it might be expected that similar findings would be observed if pain intensity was used as the clinical outcome measure.

### Physical assessment (PHY measures)

Participants in the LIG showed a reduction in symptoms with several PHY measures intended to provoke pain through movement (MCI tests, ASLR and PSLR tests, PLE test), and in aberrant movements, while similar improvements were not seen in the SIG (Table 4). These findings confirm our hypotheses concerning Class-A variables, namely that the LIG would show more improvement in these variables than the SIG as the LSEP is theoretically designed to improve these Class-A variables. We did not assess spine motion during pain-provocation tests and, as such, cannot conclude that any improvements were related to spine motion. To the authors knowledge, only one study looked at the effect of a similar LSEPs (reported as a Pilates program; twice a week during six months) on lumbar MCI tests [65]. After the intervention, lumbar MCI decreased more in the exercise group compared to the non-exercise group, which concurs with the present findings, but their MCI tests looked at signs, not symptoms. The present findings do, however, fit with the theoretical foundation for LSEPs, which aim to improve the dynamic stabilization of the spine through improvements in motor coordination [66] and, potentially, in the passive properties of the paraspinal connective tissues [67, 68]. Participants who showed improvement with LSEPs may be more likely to fit at the ‘loose control’ end of a spectrum theorized by van Dieën [69, 70], suggesting that “enhancement of muscle activity” is required to optimize the loading of spine tissues (potentially a source of pain provocation) in these participants. In other words, there would be an association among improved motor control and reduction in abnormal tissue loading, which, in turn, allows tissue repair or healing. With improved tissue healing, the patient is able to move about more freely, thus reducing disability. Effectively, it is thought that tissue repair would be possible by a better control of the relative movement between the lumbar vertebrae during the treatment, avoiding the exacerbation of the lesions at the source of the LBP and thus allowing the time necessary for this repair [71]. Unfortunately, tissue repair remains to be demonstrated and there is no way to measure these intervertebral movements, which makes this hypothesis untested.

The LIG improved more than the SIG regarding several physical tests requiring maximal performance of the participants (PPT and TME variables) (Table 3; Fig. 2). These results support the hypothesis that a LSEP improves muscular fitness, at least in terms of endurance (TME variables) and possibly in terms of muscle coordination and power (PPT variables), and that these improvements are associated with treatment success. Several studies demonstrate the positive effect of a LSEP on trunk muscle endurance [72–75]. Given that these performance measures could be influenced by pain-related psychological variables (PCS, FABQ-AP), additional correlational analyses were performed to assess this possibility. Yet, statistically significant (*P* < 0.05) but weak correlations were obtained between ∆OSW (∆ = T8-T0) and the ∆PPT (*r* = 0.21 to 0.35) and ∆TME-Back (*r* = -0.31) variables, but these correlations were only slightly decreased (by 0.03 to 0.10 points) when adjusting for ∆PCS or ∆FABQ-AP, thus rejecting this possibility. These positive results are at odds with the conclusion of a systematic review that investigated the correlation between changes observed with different performance measures (strength, mobility, endurance) and observed clinical changes (pain, perceived disabilities) during different exercise programs [76]. Their overall conclusion was that their results do not support the notion that the clinical effects of exercise therapy are directly attributable to changes in the musculoskeletal system. Strength was not directly measured in the present study but no study had related endurance to self-reported disability in this review [76]. It is possible that our approach of contrasting two very different subgroups (excluding the “clinical improvement” subgroup lying in between) may have made the results more noticeable. On the other hand, the study of our mobility measures led to the same conclusion as the Steiger, Wirth [76] review, as discussed in the next paragraph.

The lack of a significant interaction for the ROM measures (Table 3) demonstrates that although an improvement in mobility was observed during the LSEP (hip flexion and internal rotation, lumbar lateral flexion), this would not be associated with LSEP success. This is not surprising as there is no scientific evidence establishing a relationship between mobility and lumbar instability, which also explains why LSEP do not include mobility exercises. In other words, these results confirm our hypotheses concerning these Class-C variables, namely that the LIG would not show more improvement in these variables than the SIG given that the LSEP is not theoretically designed to improve these Class-C variables. However, a meta-analysis showed that trunk-focussed exercises, which encompass motor control / core stabilization, strength/resistance and flexibility of the trunk, generated improvements in the trunk (lumbar or hip) ROM that were associated with a reduction in pain and disability [77], suggesting that LSEP should be combined with trunk flexibility exercises to further improve clinical outcomes.

### Psychological assessment (PSY measures)

The magnitude of treatment effects varied as a function of scores on measures (FABQ-AP, PCS, B-IPQ) of pain-related psychological variables (Table 5; Fig. 4), revealing greater improvement in the LIG compared with the SIG. These findings confirm our hypotheses concerning these Class-B variables, namely that the LIG would show more improvement in these variables than the SIG as the LSEP is theoretically designed to indirectly improve these Class-B variables. Whereas the subgroups were equivalent at T0 in all of these cases, a gap widened between them from one measurement time to the next (T4 and T8) and stabilized at the 6-month follow-up (T34), with effect sizes exceeding the thresholds of 0.5 (average) and 0.8 (strong) in several cases. However, it is important to remember that these effects are probably not specific to a LSEP, i.e., they are possible for any type of exercise program as well as for some non-exercise interventions.

The LIG showed a greater decrease in their fears and beliefs about physical activity (FABQ-AP) and in pain catastrophizing (PCS) than the SIG. This supports the idea that LSEP allows for very gradual exposure to activity, beginning with motor control exercises, that is conducive to reducing pain and movement-related fears, as proposed by the fear-avoidance model [47] and as demonstrated for different exercise programs [78–81].

These variables have rarely been considered to study the effects of an LSEP. No effects on FABQ-AP had been detected following a LSEP inspired by the Australian approach [82, 83]; thus, the effects observed here and in another study [84] may possibly be attributable to the development of trunk muscle endurance (20–30 repetitions) that characterizes the McGill approach, as the present LSEP combines both schools of thought. Moreover, FABQ-AP was selected in one of the predictive models (model 9; FABQ-AP ≥ 6.5/24) tested to develop the CPR for success that we have recently derived [13] and the CPR for failure (FABQ-AP < 9/24) of Hicks, Fritz [11], both underlying LSEPs with a component emphasizing the development of trunk muscle endurance. This component is not trivial because lumbar stabilization exercises do not contain vigorous physical activities, nor do they contain movements other than the adoption of static lumbar postures in a prolonged and constrained (neutral spine posture) manner. Effectively, these exercises promote the control of the lumbar spine in a neutral posture, which could have been interpreted by the participants as a way to limit lumbar movements and physical activity. This, in turn, would have had the potential to heighten the fears and beliefs about physical activity, but the opposite was fortunately observed here. It thus appears that the load imposed on the lumbar structures (including the muscles) during the various postures, when maintained in such a way as to develop muscular endurance, would be sufficient to induce a shift in cognition and then, a decrease of these fears. This hypothesis is supported by the positive effects also observed on FABQ-AP [78, 85] and PCS [78], up to the 6-monh follow-up, following a Pilates exercise program, a form of exercise very comparable to the present LSEP. Taulaniemi, Kankaanpaa [85] hypothesized that this slowly progressing Pilates-type programme provides “safe” movement control, which in turn might give the participants positive experiences of movement (or decreased threat perception). Studies with longer-term follow-up would test whether this experience of success is sufficient for these participants, after eventually stopping their home exercises, to resume these exercises on their own, if these fear-avoidance beliefs are still no longer a barrier. To resume the LSEP, participants probably also need to feel competent enough to do the exercises by themselves. Unfortunately, only self-efficacy to overcome barriers to exercise was measured in the present study (not reported here as not relevant), not self-efficacy to perform the stabilization exercises per se.

Illness perceptions (B-IPQ) showed the same pattern as FABQ-PA and PCS, showing more improvement in the LIG than the SIG. Interestingly, a previous exploratory study on home exercise adherence revealed that illness perceptions was predictive of the global rating of change (GROC) measured at T8 and T34, which in turn was the main predictor of home exercise adherence measured at T34 [86]. More specifically, negative illness perceptions were associated with a lower GROC. GROC represents the fourth stage of the common-sense self-regulatory model [48], a model that appears well suited to predict adherence behaviours [87]. This fourth stage consist of the appraisal to help them decide whether they were closer to their goal (e.g., recovery or managing pain) or not.

Illness perceptions are not typically assessed in research examining the effects of exercise programs. To exclude the possibility that the observed effect was explained only by the effect also observed on pain intensity, the correlation between ∆B-IPQ (T8-T0) and ∆ODI was calculated (*n* = 105 participants) without and with adjustment for ∆NPRS, and the correlations, always significant (P < 0.001), were 0.53 and 0.41, respectively. Thus, the effect on these perceptions goes beyond symptom improvement and appears to play a role in adherence to the home-based LSEP, as discussed earlier.

### Study limitations

As mentioned above, the study design and statistical model used for this study cannot confirm the direction of causality among study variables. Another important limitation is that several dependent variables were tested, raising the risk of generating type I statistical errors (false positive). Consequently, these findings should be interpreted with caution as some of the statistically significant differences (possibly 5%) may have occurred by chance. However, the fact that the results were consistent across several measures lends them a certain credibility, although it might be expected that some measures (e.g., TME measures) or concepts (e.g. fear-avoidance and pain catastrophizing; PDI subscales and total scores) may share some common variance, which may at least partly explain these consistent findings. It’s also worth noting that most participants (98%) suffered from chronic pain (3 months or more), and that a large proportion of the sample (49%) had been experiencing pain for more than five years. Consequently, the findings of this study might not be generalizable to individuals experiencing more acute ( < less than 4 weeks) or subacute (4–12 weeks) pain. In spite of these limitations, the present study showed novel and consistent (across several measures) findings suggesting that both physical and psychological factors may have influenced the improvement in back pain related disability following a LSEP. Future research should further investigate these potential mechanisms of benefit using a RCT study design and mediation analyses, allowing more definitive conclusions to be drawn, in terms of causality, and better informing clinical intervention.

## Conclusions

The large-improvement subgroup showed more improvement than the small-improvement subgroup with regard to physical factors typically targeted by this specific exercise program as well as for psychological factors that are known to influence clinical outcomes. Taken together, these findings suggest that a lumbar stabilization exercise program may have influenced the improvement in back pain related disability (ODI) by improving different physical and psychological factors.

### Electronic supplementary material

Below is the link to the electronic supplementary material.

Supplementary Material 1

## Data Availability

The datasets used and/or analysed during the current study are available from the corresponding author on reasonable request.
