# Supplementary material for: Disability reduction following a lumbar stabilization exercise program for low back pain: large vs. small improvement subgroup analyses of physical and psychological variables
Source: BMC Musculoskelet Disord. 2024 May 4;25:358. doi: 10.1186/s12891-024-07480-4 (PMC11069239; doi:10.1186/s12891-024-07480-4)
Supplement: Supplementary file 1 — Supplementary Material 1 [file 12891_2024_7480_MOESM1_ESM.docx]

**SUPPLEMENTARY FILE**

**Detailed description of clinical outcomes (OUT), physical (PHY) and psychological (PSY) variables**

| **Measured concept**  **(category and theoretical model)** | **Test description and interrater reliability** | **Variable abbreviations (units)**  **[Class (A, B or C)]*** |
| --- | --- | --- |
| Perceived disability (OUT from the fear-avoidance model) | The original version of the Oswestry Disability Index (ODI) is a unidimensional scale with 10 items (1). Total score varies between 0 and 100%, higher scores meaning more disability. Internal consistency is satisfactory and ranges between .71 and .87 for the English version, and .87 for the French version (2). A change of 10% is considered clinically significant (3). | ODI (0-100) |
| Pain intensity (OUT from the fear-avoidance model) | An 11-point Numeric Pain Rating Scale (NPRS), anchored from 0 (no pain) to 10 (excruciating), was used to assess the average, least and worst levels of pain intensity during the last week (4). The three measures were averaged to obtain a valid composite score (5). Pain intensity at time of assessment was not measured as it can vary according to an action done before testing. A change of 2 points is considered a clinically significant change (6). | NPRS (0-10) |
| Activity-related pain (OUT from the fear-avoidance model) | The Activity-Related Pain questionnaire is one question (yes/no) assessing whether the participant has experienced increased pain during general activity or exercise: “*Do you experience increased pain during general activity or exercise?*” This variable has been associated with fear-avoidance behaviors (7). | ActRelPain (Y/N) |
| Risk of unfavorable prognosis (OUT from the fear-avoidance model) | The Subgroups for Targeted Treatment (STarT) Back Screening Tool (8) is a 9-item screening measure used to identify subgroups of patients with LBP in primary care settings based on the presence of potentially modifiable prognostic factors that may be useful in matching patients with targeted interventions (9). Psychosocial subscale scores are determined by summing items related to bothersomeness, fear, catastrophizing, anxiety, and depression (items 1, 4, 7, 8, 9). Based on the overall score (ranging from 0 to 9) and the psychosocial subscale (ranging from 0 to 5), the STarT Back categorizes patients as "high-risk" (psychosocial subscale scores ≥ 4), in which high levels of psychosocial prognostic factors are present with or without physical factors present, "medium-risk" (overall score >3; psychosocial subscale score <4), in which physical and psychosocial factors are present but not a high level of psychosocial factors, or "low-risk" (overall score 0-3), in which few prognostic factors are present. | STarT Back (0-9) |
| Fear-avoidance (PSY from the fear-avoidance model) | The Fear-Avoidance Beliefs Questionnaire (FABQ) has 16 items rated on a 7-point scale (completely disagree = 0 to completely agree = 6), with two subscales: Physical Activity (items 2, 3, 4, 5), and Work. Only the physical activity subscale (FABQ-PA) was used because participants not currently employed could not complete the Work subscale (10). The FABQ-PA is scored from 0 to 24, with higher scores indicating more fear-avoidance beliefs. There is no norm for this subscale, but previous findings suggest that a cut-off of 15 has some predictive value (11). The psychometric properties of the validation of the French version of the FABQ-PA are acceptable regarding test-retest, construct validity and responsiveness (12); the minimal clinically important change is not known but the minimum detectable change is 7 (12). | FABQ-PA (0-24)  [Class-B variable] |
| Pain catastrophizing (PSY from the fear-avoidance model) | The Pain Catastrophizing Scale (PCS) includes 13 items rated on a 5-point scale (not at all = 0 to all the time = 4) and assesses the following dimensions: rumination (4 items), amplification (3 items), and helplessness (6 items) (13). Scores above 20 and 30 correspond to moderate and high risk of chronicity, respectively (14). The psychometric qualities related to its validity, internal consistency and test-retest reliability are all acceptable. (15). The French validation reported a Cronbach’s alpha of .91 and a test-retest reliability of *r* = .85 (16), while the corresponding scores, derived from a meta-analysis (17), are 0.92 and 0.88 respectively. The total score was used in the analysis. | PCS (0-52)  [Class-B variable] |
| Psychological distress (PSY from the fear-avoidance model) | The Psychological Distress Inventory (PDI) consists of 29 items describing different feelings, thoughts or somatic perceptions related to the construct of distress, on a 4-point scale (not at all = 0 to very often = 3) (18). Because of the diversity of distress dimensions seen in different research (18-22), subscales and the total score were considered for the first objective. The 5 subscales are: anxiety, depression, somatization, anger, and cognitive problems. Higher scores mean a higher level of distress. The total score is calculated from the mean of the items, divided by three and multiplied by 100. We used the same procedure for subscales and multiplied the scores by the proportion of items for each subscale. Préville’s (19) factorial analysis was used because it isolates the somatization items from the anxiety and depression scales. Norms have been established, indicating the level of distress (23). | PDItot (total)  PDIcog (cognitive)  PDIanx (anxiety)  PDIdep (depression)  PDIang (anger)  PDIsom (somatization)  Score range of all variables: 0 – 100  [Class-B variable] |
| Illness perception (PSY from the common-sense model) | The Brief-Illness Perception Questionnaire (24) has 8 items, using a 0 - 10 rating scale and measuring the following dimensions: causes, consequences, timeline, personal control, treatment control, identity, concern, coherence and emotional representation. We used the total score, after inversion of the 3^rd^, 4^th^, and 7^th^ item scores. A high total score means negative illness perceptions. This questionnaire shows acceptable metrological qualities in patients with subacute or chronic low back pain (25, 26). | Illness perception (0-80)  [Class-B variable] |
| Habitual physical activity (PSY from the fear-avoidance model) | Habitual physical activity (HPA) during the last month was assessed with the 16-item Baecke questionnaire (27), which considers three dimensions: work activities (HPA-work: 8 items), sports activities (HPA–sport: 4 items), and leisure activities (HPA-leisure: 4 items). Most questions have a 5-category response possibility, with higher scores indicating more physical activity. The total score is on 15 points while the individual activity scores were on 5 points. It shows good test-retest reliability (ICC = 0.84-0.90, 0.71-0.83 and 0.61-0.74 for HPA-work, HPA-sport and HPA-leisure, respectively (27-29). HPA-work was not retained as some participants were not at work at the time of testing. Criterion validity against the doubly labelled water measure showed significant correlations for HPA-total score (r = 0.69) as well as HPA-work (r=0.52) and HPA-sport (r=0.55), but not for HPA-leisure (r=0.22) (30). | HPA-sport (0-5)  HPA-leisure (0-5)  [Class-B variable] |
| Treatment expectation (PSY from the common-sense model) | The Outcome Expectation for Exercise Scale (31) includes 9 items about the benefits of exercise rated on a 5-point scale (strongly disagree = 1 to strongly agree = 5). Although, there is a significant correlation with the self-efficacy for exercise scale (*r* = .66, *p* < .05), Resnick et al. (31) supports Bandura’s theory (32), and suggest they should be considered separate concepts. The total score is the mean of the 9 items on the scale. | Treatment expectation (1-5)  [Class-B variable] |

* Class-A, class-B and class-C variables refers to the strength of the theoretical link with lumbar instability. Class-A variables are the variables that are specifically (theoretically) associated with this treatment, either in direct or indirect relation to lumbar stability. Class-B variables may influence outcomes through adherence to the home exercise program; Class-C variables may be associated with any source of pain (not exclusive to lumbar instability) and consequently, to any exercise program (non-specific to the lumbar stabilization exercise program).

**Physical examination (PHY measures)**

Equipment required:

- Treatment bed
- Two manual inclinometers
- Chronometer
- Measurement tape
- Bag with weight up to 5 kg (PPT-Reach test; see below)
- Armless chair (PPT-SitStand test; see below)
- Foldable floor mat (thickness: 5 cm) for gymnastics (PPT-Rollover test; see below)
- Wooden support angled at 60° or treatment bed allowing the same (MT-Abdo test; see below)

The different tests were performed in the following sequence:

| **Measured concept**  **(test category)** | **Test description and interrater reliability** | **Data reduction and corresponding variable abbreviations (units)**  **[Class (A, B or C)]*** |
| --- | --- | --- |
| Posterior chain mobility (ROM test)  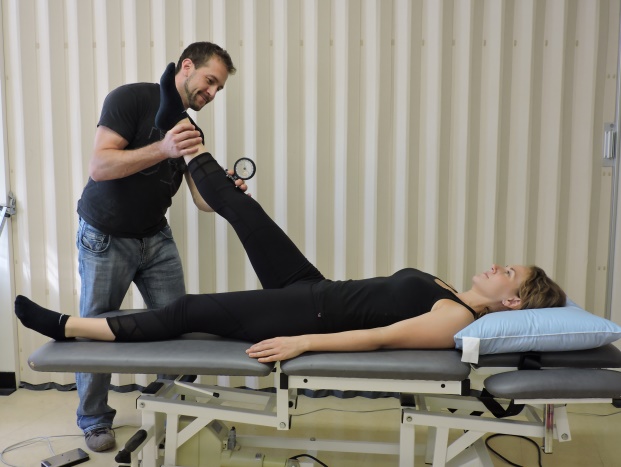 | Passive straight-leg raise (PSLR) test on L/R sides separately (33): The patient lies supine with their head relaxed on the table and is instructed to not look up. The examiner holds the patient’s foot with one hand, keeping the hip in neutral rotation. With the other hand, the examiner positions the inclinometer on the ipsilateral tibial crest, just below the tibial tubercle, setting the inclinometer to zero. The examiner then slowly raises the patient’s leg (passive movement) while holding the inclinometer in position and maintaining the patient’s knee fully extended. The movement is stopped a first time at the onset of a stretching or pain sensation (regardless of the source), and the movement is then continued up to the maximal elevation angle tolerable. The angle in degrees (ROM) is recorded in these two events. Inter-rater reliability: k = 0.67 to 0.96 (34-36). | Minimal L/R scores retained:  PSLR-Pain ROM-min (°)  PSLR-Max ROM-min (°)  [Class-C variable] |
| Load transfer of lumbo-pelvic area  (ASLR test)  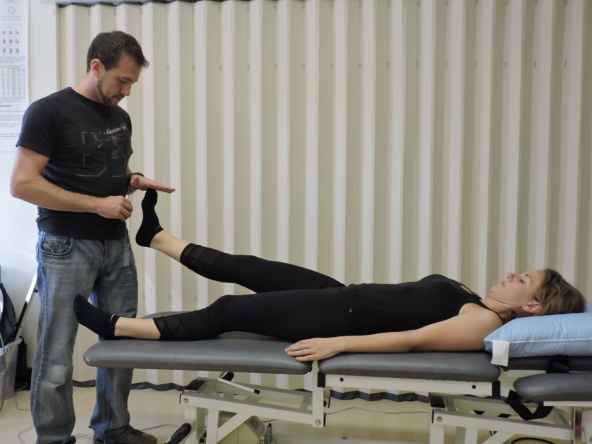 | Active straight-leg raise (ASLR) on L/R sides separately (37): The patient lies supine with their legs straight and their feet 20 cm apart. They are instructed to raise one foot 20 cm above the table without bending their knee. The patient is then asked to score a perceived effort to perform the test on a 6-point scale: not difficult at all (0), minimally difficult (1), somewhat difficult (2), fairly difficult (3), very difficult (4), or unable to perform (5). The patient is then asked whether they felt pain: no (0) or yes (1). Reliability: k = 0.70 to 0.77 (36-38) but lower reliability (k = 0.53) has been reported (39). | Maximal L/R scores retained:  ASLR-Act/5-max (/5)  ASLR-Pain-max (0 or 1)  [Class-A variable] |
| Lumbar lordosis (posture test)  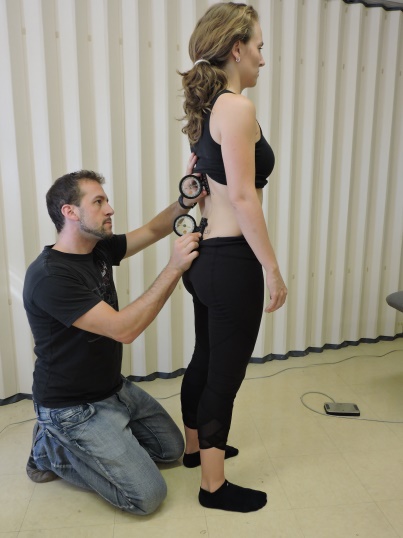 | Lumbar lordosis (40): The participant is asked to stand in a relaxed posture with the heels about shoulder width apart, hands hanging freely by the side and eyes looking forward. The lumbar lordosis is measured with inclinometer recordings at T12-L1 and L5-S1.  Intra-rater reliability (3 days interval): ICC = 0.95 (40). Inter-rater reliability: ICC = 0.97 (41). | Lordosis (°)    [Class-A variable] |
| Pelvis and lumbar flexion ROM  (ROM test)  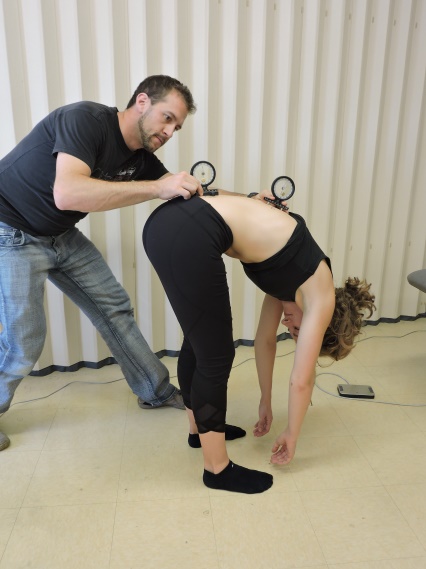 | Pelvis and lumbar flexion ROM (42): The patient is asked to stand in a comfortable position with their arms hanging loose at their sides. From this position, the patient is instructed to perform maximum trunk flexion, keeping their knees straight, especially at the end of movement. Lumbar angle at maximum trunk flexion is measured from inclinometers on the spinous processes of T12 and S1. Intra-rater reliability (3 days interval): ICC = 0.87 (40). Inter-rater reliability: ICC = 0.87 to 0.89 (41, 42). | PelvisFlx-ROM (°)  LumbFlx-ROM (T12 minus S1 angles) (°)  [Class-C variable] |
| Lumbar lateral flexion ROM (ROM test)  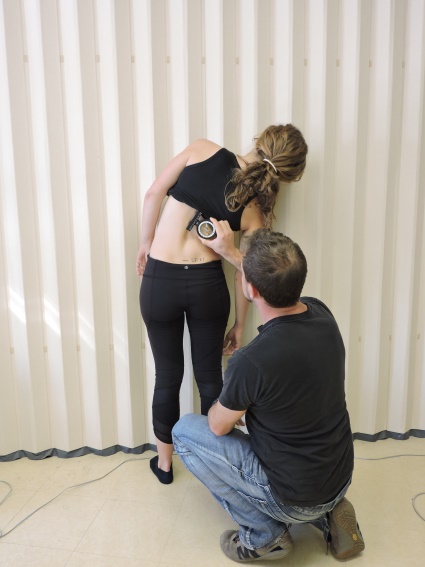 | Lumbar lateral flexion ROM (41): The patient stands with the inclinometer aligned vertically in line with the spinous processes of T9 and T12. The patient is asked to lean to one side, as far as possible, by reaching their fingertips down the side of their thigh. The examiner should support the patient’s shoulder with one hand and make sure that the patient does not flex forward or twist around and that both feet stay flat on the ground. Intra-rater reliability (3 days interval): ICC = 0.92 to 0.96 (40). Inter-rater reliability: ICC = 0.92 to 0.95 (41). | Maximal L/R score retained:  LumbLatFlx-ROM-Min (°)  [Class-C variable] |
| Lumbar instability (LSI test)  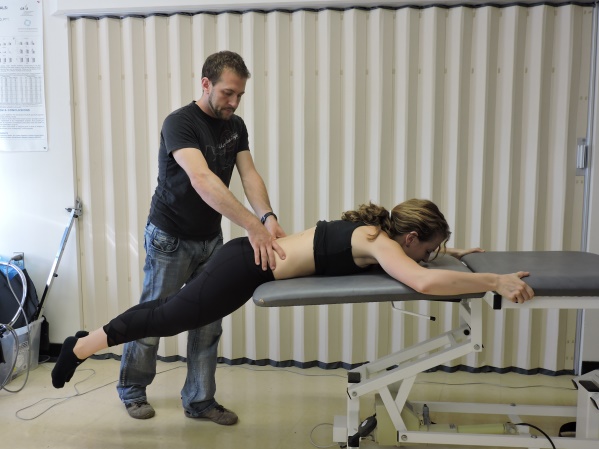 | Prone instability test (43): The patient lies prone with their upper body on the examining table and legs over the edge and feet resting on the floor. The examining table is positioned specific to the patient, such that when the patient is standing, the table height is set at the lower one third of the patient’s thigh. The patients’ hips are positioned at the end of the table, so that their entire legs are off the table. The knee should be slightly flexed and relaxed and feet resting on the floor to support the legs’ weight and make sure that the lumbar spine is not in traction. While the patient rests in this position, the examiner applies posterior-anterior pressure to the spinous process of each vertebra of the lumbar spine, starting from L1. If no pain is reported, the test is coded as negative and the remainder of the test does not need to be completed. If pain is reported, the examiner removes the pressure instructs the patient to lift their feet off the floor (the patient may hold table using bent elbows at head level to maintain position). The posterior-anterior pressure is reapplied in this second position. If pain is not reproduced in this second position, the test is positive. If pain is present with passive provocation testing in both the resting position and with the feet actively held off the floor, the test is negative. If the pain level decreases, but does not disappear in the second position, the test also is negative. Inter-rater reliability: k = 0.67 to 0.87 (39, 44-47), but lower reliability results (k = 0,27-0.54) were also obtained elsewhere (48, 49). A critical appraisal of these studies concluded that this test shows good interrater reliability (50). A more recent review led to a moderate recommendation for the use of this test (51). | ProneIT (0/1; 1 if positive)  [Class-A variable] |
| Lumbar instability (LSI test)  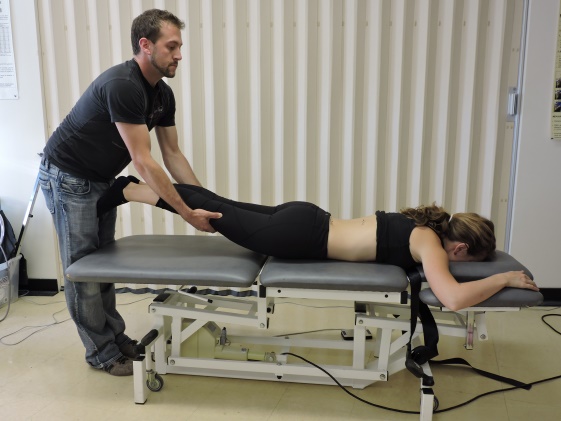 | Passive lumbar extension (PLE) test (52): With the patient prone, the examiner holds the patient’s feet and passively elevates both lower extremities, applying gentle traction and maintaining the knees extended, stopping when movement is seen in the lumbar spine. The test is positive if the patient reports pain or a strong abnormal sensation in the lumbar region (e.g., low back feeling “very heavy” or felt to be “coming off”) which disappears when the legs are returned to the initial position. The test is otherwise negative, even in the case of mild abnormal sensations, such as paraesthesia. Inter-rater reliability: k = 0.76 (39) or 0.46 (47). A critical appraisal of these studies reached no conclusion about the test’s interrater reliability (50). | PLE (0/1; 1 if positive)  [Class-A variable] |
| Motor control impairments (MCI tests)  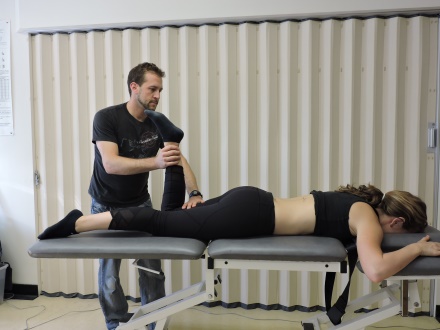  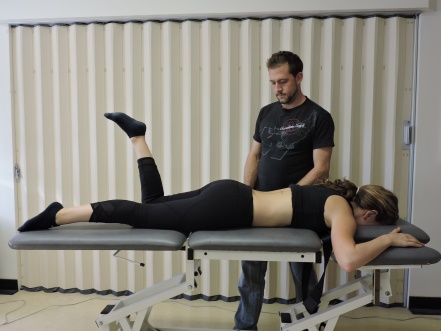  Passive and active knee flexion | MCI looking at symptoms (increase: 1, decrease: -1, the same: 0) during prone knee flexion on L/R sides separately (and in active and passive modes separately) (53): The patient lies prone with the hips positioned in neutral abduction/adduction and neutral rotation. The patient’s arms are positioned at their sides. If the table has no face hole, their head is turned to whichever side is most comfortable. If pain level in this position is more than usual, the examiner assists the patient to adjust their position with one or two pillows under the belly, noting any adjustment. For the active test, the patient flexes one knee to 90° and then returns it to the starting position. For the passive test, this movement is performed by the examiner. Each test (active and passive) is performed separately with each lower extremity. The symptoms with the test movement are compared to the patient’s symptoms in prone. Inter-rater reliability (symptoms): k = 0.87 (54). | The maximal L/R score were retained (values of -1, 0 or 1), then dichotomized as follows:  0 if -1 or 0 and 1 if 1.  MCI-KneeF-Pas-Max (0/1; 1 if positive)  MCI-KneeF-Act-Max (0/1; 1 if positive)  [Class-A variable] |
| Motor control impairments (MCI tests)  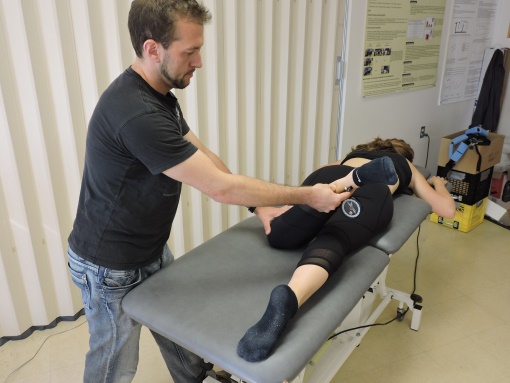  Passive hip internal rotation  (no picture for the active test)  Passive hip external rotation: Same picture as above but the hip was moved in external rotation. | MCI looking at symptoms (increase: 1, decrease: -1, the same: 0) during prone hip rotation on L/R sides separately (and in active and passive modes separately) (53): The patient lies prone with the hips positioned in neutral abduction/adduction and neutral rotation. The patient’s arms are positioned at their sides. If the table has no face hole, their head is turned to whichever side is most comfortable. If pain level in this position is more than usual, the examiner assists the patient to adjust their position with one or two pillows under the belly, noting any adjustment. The examiner then passively flexes one of the patient’s knees to 90°. For the active test, the patient is asked to bring their foot out to the side, as far as possible, by rotating their hip (internal rotation), and then back across the midline, as far as possible (external rotation). For the passive test, this movement is performed by the examiner. Each test (active and passive) is performed separately with each lower extremity. The symptoms with the test movement are compared to the patient’s symptoms in prone. Inter-rater reliability (symptoms): k = 0.98 (54).  **Addendum: In the sitting position and using an inclinometer placed on the lateral side of the lower leg, the evaluator measures passive ROM of each hip, from the neutral position, for internal and external rotation.** Inter-rater reliability of internal rotation (ICC = 0.74-0.93) across left and right sides and across days) and external rotation (ICC = 0.85-0.93) across left and right sides and across days) (55). | For symptoms, the maximal L/R scores were retained (values of -1, 0 or 1), then dichotomized as follows: 0 if -1 or 0 and 1 if 1.  MCIP-HipIR-Pas-max (0/1; 1 if positive)  MCIP-HipIR-Act-max (0/1; 1 if positive)  MCIP-HipER-Pas-max (0/1; 1 if positive)  MCIP-HipER-Act-max (0/1; 1 if positive)  [Class-A variable]  For ROM, the minimal L/R scores were retained  MCIP-HipIR-Pas-ROM-min (°)  MCIP-HipER-Pas-ROM-min (°)  [Class-C variable] |
| Motor control impairments (MCI tests)  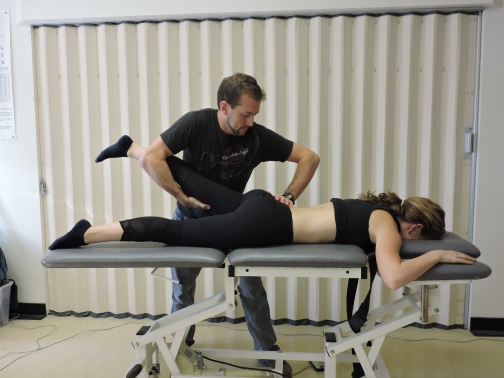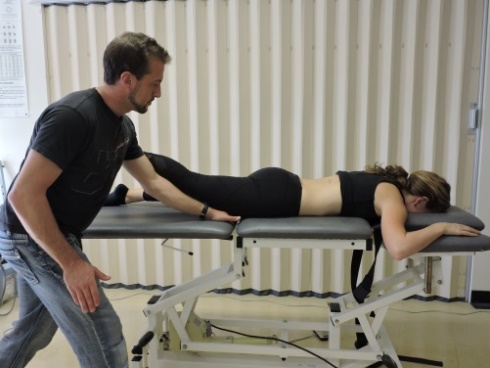  Passive and active hip extension | MCI looking at symptoms (increase: 1, decrease: -1, the same: 0) during prone hip extension on L/R sides separately (and in active and passive modes separately) (53): The patient lies prone with the hips positioned in neutral abduction/adduction and neutral rotation. The patient’s arms are positioned at their sides. If the table has no face hole, their head is turned to whichever side is most comfortable. If pain level in this position is more than usual, the examiner assists the patient to adjust their position with one or two pillows under the belly, noting any adjustment. For the active test, the patient is asked to lift one thigh off the table while keeling their knee straight, so that the examiner can pass their hand under it (available hip extension ROM, up to 10°). For the passive test, the movement is performed by the examiner. Each test (active and passive) is performed separately with each lower extremity. The symptoms with the test movement are compared to the patient’s symptoms in prone. Inter-rater reliability (symptoms): k = 0.97 (54). | The maximal L/R score were retained (values of -1, 0 or 1), then dichotomized as follows:  0 if -1 or 0 and 1 if 1.  MCIP-HipE-Pas-max (0/1; 1 if positive)  MCIP-HipE-Act-max (0/1; 1 if positive)  [Class-A variable] |
| Motor control impairments (MCI tests)  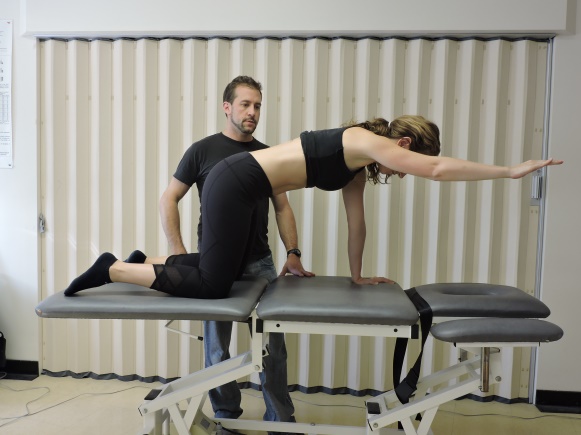  Active arm lifting | MCI looking at symptoms (increase: 1, decrease: -1, the same: 0) during standardized quadruped kneeling and arm lifting, on L/R sides separately (in active mode only), as described by (56): The patient assumes a four-point-kneeling position: lumbar spine parallel to supporting surface, without lumbar region rotation, pelvic rotation, or lateral pelvic tilt; hips at 90° flexion, aligned over the knees, in neutral rotation and abduction/adduction; ankles plantarflexed; and shoulders at 90° of flexion. The patient is asked to lift one arm toward the side of their head, to a maximum of 180° flexion, while keeping the elbow extended. The test is then repeated on the contralateral side The symptoms with each test movement are compared to the patient’s symptoms in the initial quadruped position. Inter-rater reliability (symptoms): k = 0.89 (54). | The maximal L/R score were retained (values of -1, 0 or 1), then dichotomized as follows:  0 if -1 or 0 and 1 if 1.  MCI4-ShoF-Act-max (0/1; 1 if positive)  [Class-A variable] |
| Motor control impairments (MCI tests)  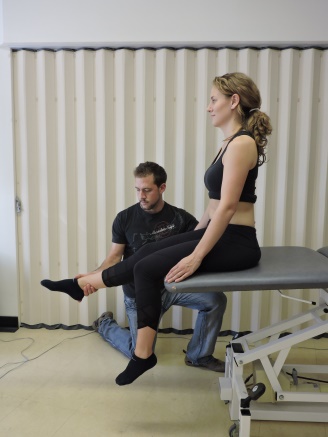  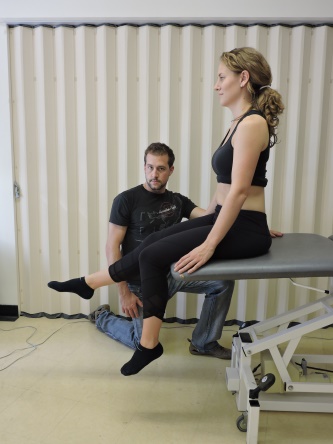  Passive and active knee extension | MCI looking at symptoms (increase: 1, decrease: -1, the same: 0) during sitting knee extension on L/R sides separately (and in active and passive modes separately) (57): The patient begins in sitting: lumbar spine flat; hips at 90° of flexion, neutral abduction/adduction and rotation, femurs fully supported; lower extremities relaxed with feet supported. For the active test, the patient is asked to straighten one knee as far as possible and return to the starting position. For the passive test, the movement is performed by the examiner. Each test (active and passive) is performed separately with each lower extremity and the examinator makes sure that the lumbar lordosis is maintained. The symptoms with each test movement are compared to the patient’s symptoms in sitting with the lumbar spine positioned in a neutral alignment. Inter-rater reliability (symptoms): k = 1.00 (54). | The maximal L/R score were retained (values of -1, 0 or 1), then dichotomized as follows:  0 if -1 or 0 and 1 if 1.  MCIT-KneeE-Pas-max (0/1; 1 if positive)  MCIT-KneeE-Act-max (0/1; 1 if positive)  [Class-A variable] |
| Motor control impairments (MCI tests)  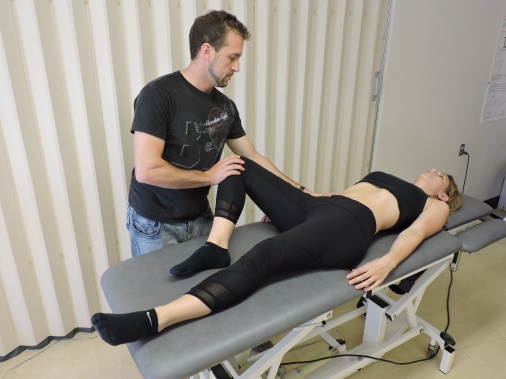  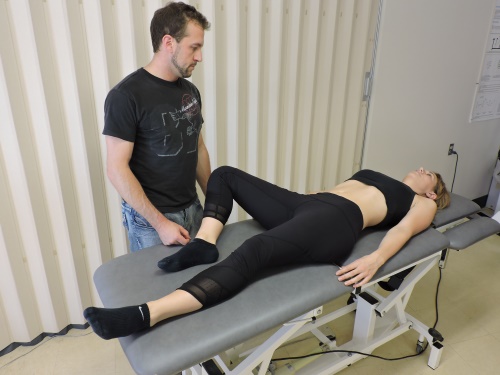  Passive and active tests | MCI looking at symptoms (increase: 1, decrease: -1, the same: 0) during hip flexion, abduction, external rotation on L/R sides separately (and in active and passive modes separately) (53): The patient lies supine with their arms at their sides, legs aligned parallel to the trunk, one lower extremity extended, and the other flexed at the hip and knee so that the foot is flat on the table with the heel aligned with the opposite patella. For the active test, the patient is asked to bring their knee out to the side (hip abduction and external rotation) with the lower extremity that was flexed. For the passive test, the movement is performed by the examiner. The symptoms with the test movement were compared to the patient’s symptoms when lying in the starting position for the test movement. Inter-rater reliability (symptoms): k = 0.98 (54). | The maximal L/R score were retained (values of -1, 0 or 1), then dichotomized as follows:  0 if -1 or 0 and 1 if 1.  MCIS-HipAR-Pas-max (0/1; 1 if positive)  MCIS-HipAR-Act-max (0/1; 1 if positive)  [Class-A variable] |
| Ligamentous laxity  (LSI test) | Beighton scale (45): Measures based on visual observation; if not clear, the test is negative). Four tests are assessed on the right and left side and a point is given for each test the patient can perform. The bilateral tests are:   - Passive hyperextension of the elbow greater than 10° - Passive hyperextension of the fifth finger to greater than 90° - Passive abduction of the thumb to contact the forearm - Passive hyperextension of the knees greater than 10°   The final test is the ability to flex the trunk and place both hands flat on the floor without flexing the knees. Each test scored negative (0) or positive (1) producing a total score from 0 to 9, higher indicating greater laxity. Inter-rater reliability: k = 0.72 to 0.79 (44, 45). | Beighton (/9)  [Class-A variable] |
| Aberrant movements (LSI test)  No picture available | Aberrant movements (45): From standing, the patient is asked to flex the trunk forward, as far as possible, while the examiner watches for any of the following:   - Painful arc in flexion: symptoms felt during the movement at a particular point in the motion that are not present before or after this point - Painful arc on return: symptoms occur only during return from the flexed to the erect position - Gower sign (thigh climbing): pushing on the thighs or another surface with the hands for assistance during return from the flexed to the erect position - Instability catch: any sudden acceleration or deceleration of trunk movement or movement occurring outside the primary plane of motion (e.g., Lateral bending or rotation) - Reversal of lumbopelvic rhythm: on attempting to return from the flexed position, the patient bends the knees and shifts the pelvis anteriorly before returning to the erect position.   Scored as positive is any of the above is observed. Only one trial is allowed.  Inter-rater reliability: k = 0.60 to 0.97 (39, 45-47, 58), but lower reliability results (k = -0.07) were also obtained elsewhere (44). A critical appraisal of these studies concluded that this test has moderate to good interrater reliability (50). | Abe-Mvt (0/1; 1 if positive)  [Class-A variable] |
| Physical performance test (PPT)  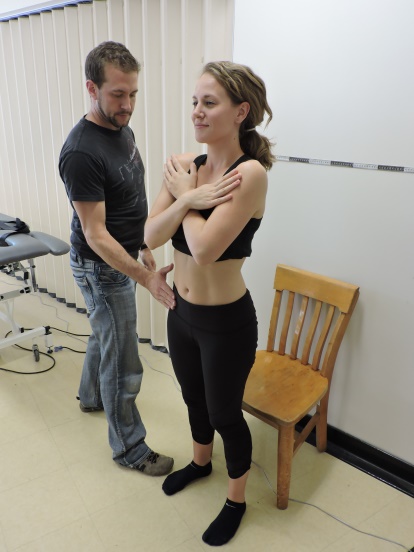 | Repeated sit-to-stand (59): “*This task uses a standard armless chair and requires the patient to stand from a sitting position and, as fast as possible, to return to the seated position 5 times.*” Additional constraints: Arms should be crossed in front and a target provided (pelvis position when standing) to ensure that complete standing is achieved (knees and/or trunk at vertical). Inter-rater reliability: ICC = 0.99 (60). Please note that the task was done only once (not the average time of 2 trials), but participants first practiced one or two repetitions, at a slow pace, to ensure proper execution. | PPT-SitStand (sec)  [Class-A variable] |
| Physical performance test (PPT)  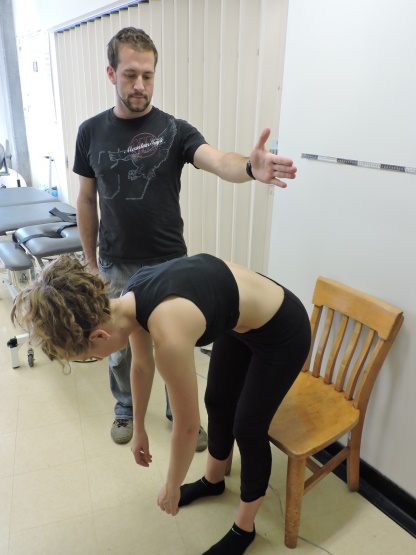  Note: The chair is not used in this test | Repeated trunk flexion (59): “*This task requires the patient to bend forward (as if touching his/her toes) and, as fast as tolerated, return to upright standing 5 times.*” Inter-rater reliability: ICC = 0.99 (60). Please note that the task was done only once (not the average time of 2 trials), but participants first practiced one or two repetitions, at a slow pace, to ensure proper execution. Slight bending of the knees was allowed during the test. | PPT-Flexion (sec)  [Class-A variable] |
| Physical performance test (PPT)  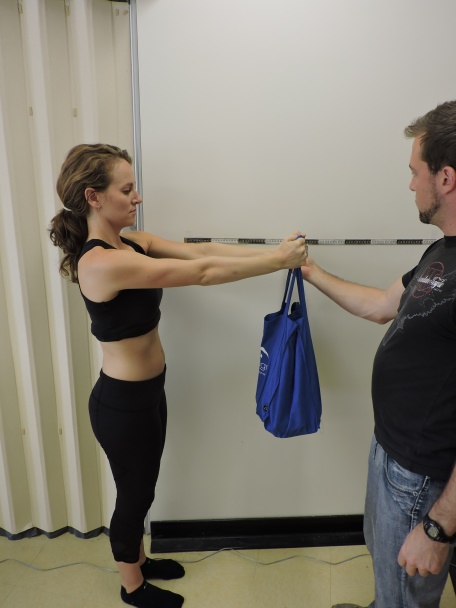 | Loaded reach (59): “*To perform this task, a light wooden bar with sandbag weights of 5% of the person’s body weight (not exceeding 4.5kg) is held in both hands at shoulder height close to the body. Patients are required to reach forward as far as possible. The distance reached is the resulting score.*” Inter-rater reliability: ICC = 0.99 (60).  To further standardize this test, a tape measure is taped horizontally to the wall at the height of the acromion, and then the participant is positioned so that the lateral edge of the acromion is placed at 0 cm, very close to the wall. Then, the task is practiced once, with no load in the hands (fists closed), to ensure proper execution, and to measure the distance of the first knuckles when both arms are totally extended. This distance is recorded as the arm length. The distance measured during loaded reach was then divided by arm length to obtain a ratio (normalized) variable. | NPPT-Reach (ratio)  [Class-A variable] |
| Physical performance test (PPT)  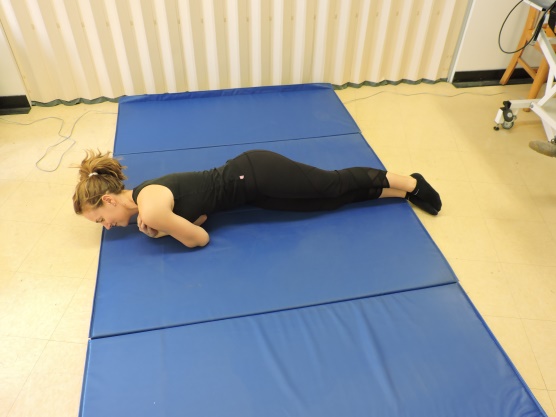 | 360° rollover on L/R sides separately (59): “*This task requires the patient to lie supine and then to roll over a full 360° as fast as possible. After a brief pause, they roll 360° in the opposite direction. The order of direction is randomized*. *Each direction of rolling is timed separately and the average time taken is the resulting score.”* In the present study, this test was carried out on a foldable gymnastics floor mat (thickness: 5 cm). Intra-rater (within-day) reliability: ICC = 0.89 (59). Inter-rater reliability: ICC = 0.81 (59). The task was practiced once (1 or 2 cycles), at a slow pace, to ensure proper execution. | Max L/R score retained:  PPT-Rollover-max (sec)  [Class-A variable] |
| Trunk muscle endurance (TME test)  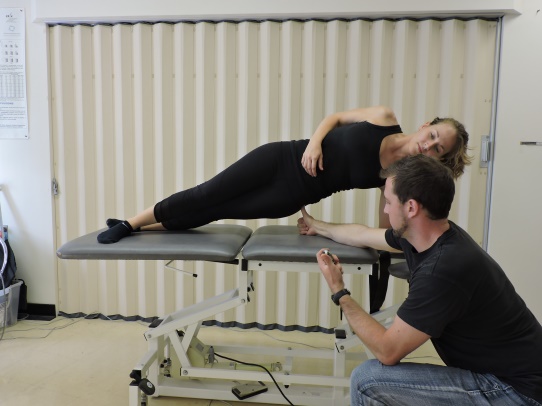 | Side bridge endurance on L/R sides separately (61): The lateral musculature is tested with the person lying in the full side-bridge position (on the treatment table): both legs extended, top foot in front of the bottom foot. Patients support themselves on one elbow and on their feet while lifting their hips off the floor to create a straight line over their body length. Failure occurs when the person no longer maintains the posture with the back straight and the hip returns to the ground, or if the person no longer maintains the light contact against the therapist's thumb that marks the hip's initial height. Intra-rater reliability: reliability coefficient of 0.99 (5 days interval) and 0.96 (8 weeks interval) (61).  Note: The uninvolved arm is simply resting along the trunk as holding it across the chest with the hand placed on the opposite shoulder was too difficult. | Minimal L/R scores retained:  TME-Side-min (sec)  [Class-A variable] |
| Trunk muscle endurance (TME test)  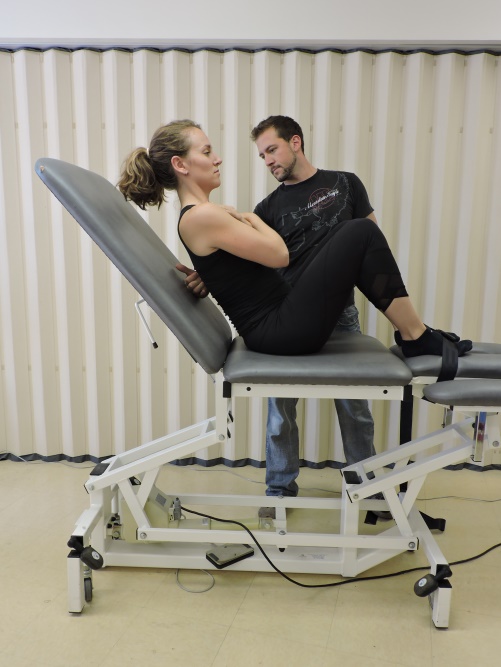 | Trunk flexor endurance (61): Testing endurance of the flexors (esp. rectus abdominis) begins with the person in a sit-up posture with the shoulder blades resting against a wooden support angled at 60° from the floor and the pelvis being spaced from the support by a fist. Both knees and hips are flexed 90°, the arms are folded across the chest with the hands placed on the opposite shoulder, and toes are secured under toe straps. The patient looks at the angle between the ceiling and the wall in front of them, during the whole test, to ensure a good cervical spine alignment. To begin, the patient is asked to lift their shoulder blades from the support until the evaluator can place two fingers between the support and the thoracic spine (T6). The patient holds this isometric posture for as long as possible while the evaluator observes for a good alignment of the cervical spine. Failure occurs when the patient cannot prevent putting pressure on the evaluator’s fingers (between the support and the thoracic spine). Intra-rater reliability: reliability coefficient of 0.97 (5 days interval) and 0.93 (8 weeks interval) (61). | TME-Abdominals (sec)  [Class-A variable] |
| Trunk muscle endurance (TME test)  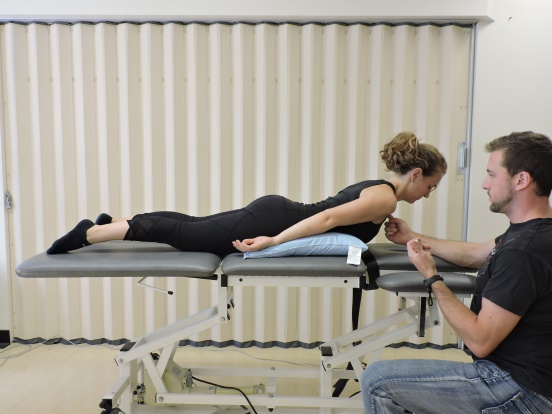 | Back extensor endurance (62): The patient is asked to lie prone while holding the sternum off the floor. A small pillow is placed under the lower abdomen to decrease the lumbar lordosis. The patient is asked to maintain their maximum flexion of cervical spine, with pelvic stabilization through gluteal muscle contraction. The patient is asked to maintain the original position for as long as possible, up to 5 minutes. Failure occurs when the patient cannot prevent putting pressure on the evaluator’s fingers positioned just below the sternoclavicular joint, but without touching it. Intra-rater reliability (3 days interval): ICC = 0.93 to 0.97 across healthy and CLBP men and women groups (62). | TME-Back (sec)  [Class-A variable] |

**Act** : active; **ASLR**: active straight leg rising; **HipAR**: hip abduction + rotation; **HipER**: hip external rotation; **HipE**: hip extension; **HipIR**: hip internal rotation; **KneeE**: knee extension; **KneeF**: knee flexion; **L/R** : left and right; **LSI**: lumbar segmental instability; **MCI**: motor control impairment; **MCI4**: motor control impairment in quadruped kneeling baseline position; **MCIP**: motor control impairment in prone baseline position; **MCIS**: motor control impairment in supine baseline position; **MCIT**: motor control impairment in sitting baseline position; **TME**: trunk muscle endurance; **Pas**: passive; **PLE**: Passive lumbar extension; **PPT**: physical performance test; **PSLR**: passive straight leg rising; **ROM**: range of motion; **ShoF**: shoulder flexion.

* Class-A, class-B and class-C variables refers to the strength of the theoretical link with lumbar instability. Class-A variables are the variables that are specifically (theoretically) associated with this treatment, either in direct or indirect relation to lumbar stability. Class-B variables may influence outcomes through adherence to the home exercise program; Class-C variables may be associated with any source of pain (not exclusive to lumbar instability) and consequently, to any exercise program (non-specific to the lumbar stabilization exercise program).

**REFERENCES**

1. Fairbank JCT, Couper J, Davies J, O'Brien J. The Oswestry low back pain disability questionnaire. Physiotherapy. 1980;66(8):271-3.

2. Vogler D, Paillex R, Norberg M, de Goumoëns P, Cabri J. Validation transculturelle de l’Oswestry disability index en français / Cross-cultural validation of the Oswestry disability index in French. Annales de Réadaptation et de Médecine Physique. 2008;51(5):379-85.

3. Ostelo RWJG, Deyo RA, Stratford P, Waddell G, Croft P, Von Korff M, et al. Interpreting change scores for pain and functional status in low back pain: towards international consensus regarding minimal important change. Spine (Phila Pa 1976). 2008;33(1):90-4.

4. Cleeland CS, Ryan KM. Pain assessment: global use of the Brief Pain Inventory. Ann Acad Med Singap. 1994;23(2):129-38.

5. Jensen MP, Turner LR, Turner JA, Romano JM. The use of multiple-item scales for pain intensity measurement in chronic pain patients. Pain. 1996;67(1):35-40.

6. Ostelo RW, Deyo RA, Stratford P, Waddell G, Croft P, Von Korff M, et al. Interpreting change scores for pain and functional status in low back pain: towards international consensus regarding minimal important change. Spine. 2008;33(1):90-4.

7. Damsgard E, Thrane G, Anke A, Fors T, Røe C. Activity-related pain in patients with chronic musculoskeletal disorders. Disabil Rehabil. 2010;32(17):1428-37.

8. Hill JC, Dunn KM, Lewis M, Mullis R, Main CJ, Foster NE, et al. A primary care back pain screening tool: identifying patient subgroups for initial treatment. Arthritis Rheum. 2008;59(5):632-41.

9. Delitto A, George SZ, Van Dillen L, Whitman JM, Sowa G, Shekelle P, et al. Low Back Pain. Journal of Orthopaedic & Sports Physical Therapy. 2012;42(4):A1-A57.

10. Waddell G, Newton M, Henderson I, Somerville D, Main CJ. A Fear-Avoidance Beliefs Questionnaire (FABQ) and the role of fear-avoidance beliefs in chronic low back pain and disability. Pain. 1993;52(2):157-68.

11. Burton AK, Waddell G, Tillotson KM, Summerton N. Information and advice to patients with back pain can have a positive effect. A randomized controlled trial of a novel educational booklet in primary care. Spine. 1999;24(23):2484-91.

12. Chaory K, Fayad F, Rannou F, Lefevre-Colau M-M, Fermanian J, Revel M, et al. Validation of the French Version of the Fear Avoidance Belief Questionnaire. Spine (Phila Pa 1976). 2004;29(8):908-13.

13. Sullivan MJL, Bishop SR, Pivik J. The Pain Catastrophizing Scale: Development and validation. Psychol Assess. 1995;7(4):524-32.

14. Sullivan MJL. User Manual/PCS 2009 [Available from: <http://www.sullivan-painresearch.mcgill.ca/pcs1.php>.

15. Pedler A. The Pain Catastrophising Scale. Journal of physiotherapy. 2010;56(2):137.

16. French DJ, Noël M, Vigneau F, French JA, Cyr CP, Evans RT. L'Échelle de dramatisation face à la douleur PCS-CF: Adaptation canadienne en langue française de l'échelle"Pain Catastrophizing Scale". Canadian Journal of Behavioural Science/Revue canadienne des sciences du comportement. 2005;37(3):181-92.

17. Wheeler CHB, Williams ACC, Morley SJ. Meta-analysis of the psychometric properties of the Pain Catastrophizing Scale and associations with participant characteristics. Pain. 2019;160(9):1946-53.

18. Ilfeld FWJ. Further validation of a psychiatric symptom index in a normal population. Psychological reports. 1976;39(3f):1215-28.

19. Préville M, Potvin L, Boyer R. The structure of psychological distress. Psychol Rep. 1995;77(1):275-93.

20. Turner JA, Shortreed SM, Saunders KW, LeResche L, Berlin JA, Korff MV. Optimizing prediction of back pain outcomes. Pain. 2013;154(8):1391-401.

21. Dionne CE. Psychological distress confirmed as predictor of long-term back-related functional limitations in primary care settings. J Clin Epidemiol. 2005;58(7):714-8.

22. Main CJ. The modified somatic perception questionnaire (MSPQ). Journal of psychosomatic research. 1983;27(6):503-14.

23. Préville M, Boyer R, Potvin L, Perreault C, Légaré G. La détresse psychologique: détermination de la fiabilité et de la validité de la mesure utilisée dans l’enquête Santé Québec. Québec: Direction des communications, Ministère de la santé et des services sociaux; 1992.

24. Broadbent E, Petrie KJ, Main J, Weinman J. The Brief Illness Perception Questionnaire. J Psychosom Res. 2006;60(6):631-7.

25. Løchting I, Garratt A, Storheim K, Werner E, Grotle M. Evaluation of the Brief Illness Perception Questionnaire in Sub-Acute and Chronic Low Back Pain Patients: Data Quality, Reliability and Validity. Journal of pain & relief. 2013;2(3):doi: 10.4172/2167-0846.1000122.

26. Ng TS. Brief Illness Perception Questionnaire (Brief IPQ). Journal of Physiotherapy. 2012;58(3):202.

27. Baecke JAH, Burema J, Frijters JER. A short questionnaire for the measurement of habitual physical activity in epidemiological studies. The American Journal of Clinical Nutrition. 1982;36(5):936-42.

28. Jacob T, Baras M, Zeev A, Epstein L. Low back pain: reliability of a set of pain measurement tools. Arch Phys Med Rehabil. 2001;82(6):735-42.

29. Carvalho FA, Morelhão PK, Franco MR, Maher CG, Smeets RJEM, Oliveira CB, et al. Reliability and validity of two multidimensional self-reported physical activity questionnaires in people with chronic low back pain. Musculoskeletal Science and Practice. 2017;27:65-70.

30. Philippaerts RM, Westerterp KR, Lefevre J. Doubly labelled water validation of three physical activity questionnaires. International Journal of Sports Medicine. 1999;20(5):284-9.

31. Resnick B, Zimmerman SI, Orwig D, Furstenberg A-L, Magaziner J. Outcome Expectations for Exercise Scale: Utility and Psychometrics. The journals of gerontology Series B, Psychological sciences and social sciences. 2000;55(6):S352-S6.

32. Bandura A. Self-efficacy: The exercise of control. New York: W. H. Freeman; 1997. 604 p.

33. Waddell G. Clinical evaluation of disability in low back pain. In: Frymoyer JW, editor. The adult spine: Principles and practice. 2nd ed. Philadelphia: Lippincott-Raven; 1997. p. 171-83.

34. Bertilson BC, Bring J, Sjoblom A, Sundell K, Strender LE. Inter-examiner reliability in the assessment of low back pain (LBP) using the Kirkaldy-Willis classification (KWC). Eur Spine J. 2006;15(11):1695-703.

35. McCarthy CJ, Gittins M, Roberts C, Oldham JA. The reliability of the clinical tests and questions recommended in international guidelines for low back pain. Spine. 2007;32(8):921-6.

36. Waddell G, Somerville D, Henderson I, Newton M. Objective clinical evaluation of physical impairment in chronic low back pain. Spine. 1992;17(6):617-28.

37. Mens JM, Vleeming A, Snijders CJ, Koes BW, Stam HJ. Reliability and validity of the active straight leg raise test in posterior pelvic pain since pregnancy. Spine (Phila Pa 1976). 2001;26(10):1167-71.

38. Roussel NA, Nijs J, Truijen S, Smeuninx L, Stassijns G. Low back pain: clinimetric properties of the Trendelenburg test, active straight leg raise test, and breathing pattern during active straight leg raising. J Manipulative Physiol Ther. 2007;30(4):270-8.

39. Rabin A, Shashua A, Pizem K, Dar G. The interrater reliability of physical examination tests that may predict the outcome or suggest the need for lumbar stabilization exercises. J Orthop Sports Phys Ther. 2013;43(2):83-90.

40. Ng JK, Kippers V, Richardson CA, Parnianpour M. Range of motion and lordosis of the lumbar spine: reliability of measurement and normative values. Spine. 2001;26(1):53-60.

41. Waddell G, Somerville D, Henderson I, Newton M. Objective clinical evaluation of physical impairment in chronic low back pain. Spine (Phila Pa 1976). 1992;17(6):617-28.

42. Saur PMM, Ensink RBM, Frese K, Seeger D, Hildebrandt J. Lumbar range of motion: Reliability and validity of the inclinometer technique in the clinical measurement of trunk flexibility,. Spine. 1996;21(11):1332-8.

43. Stanton TR, Fritz JM, Hancock MJ, Latimer J, Maher CG, Wand BM, et al. Evaluation of a treatment-based classification algorithm for low back pain: a cross-sectional study. Phys Ther. 2011;91(4):496-509.

44. Fritz JM, Piva SR, Childs JD. Accuracy of the clinical examination to predict radiographic instability of the lumbar spine. Eur Spine J. 2005;14(8):743-50.

45. Hicks GE, Fritz JM, Delitto A, Mishock J. Interrater reliability of clinical examination measures for identification of lumbar segmental instability. Arch Phys Med Rehabil. 2003;84(12):1858-64.

46. Puntumetakul R, Yodchaisarn W, Emasithi A, Keawduangdee P, Chatchawan U, Yamauchi J. Prevalence and individual risk factors associated with clinical lumbar instability in rice farmers with low back pain. Patient preference and adherence. 2015;9:1.

47. Alyazedi FM, Lohman EB, Wesley Swen R, Bahjri K. The inter-rater reliability of clinical tests that best predict the subclassification of lumbar segmental instability: structural, functional and combined instability. Journal of Manual & Manipulative Therapy. 2015;23(4):197-204.

48. Schneider M, Erhard R, Brach J, Tellin W, Imbarlina F, Delitto A. Spinal palpation for lumbar segmental mobility and pain provocation: an interexaminer reliability study. Journal of Manipulative and Physiological Therapeutics. 2008;31(6):465-73.

49. Ravenna MM, Hoffman SL, Van Dillen LR. Low interrater reliability of examiners performing the prone instability test: a clinical test for lumbar shear instability. Arch Phys Med Rehabil. 2011;92(6):913-9.

50. Denteneer L, Stassijns G, De Hertogh W, Truijen S, Van Daele U. Inter- and Intrarater Reliability of Clinical Tests Associated With Functional Lumbar Segmental Instability and Motor Control Impairment in Patients With Low Back Pain: A Systematic Review. Arch Phys Med Rehabil. 2017;98(1):151-64 e6.

51. Stolz M, von Piekartz H, Hall T, Schindler A, Ballenberger N. Evidence and recommendations for the use of segmental motion testing for patients with LBP - A systematic review. Musculoskelet Sci Pract. 2019;45:102076.

52. Kasai Y, Morishita K, Kawakita E, Kondo T, Uchida A. A new evaluation method for lumbar spinal instability: passive lumbar extension test. Phys Ther. 2006;86(12):1661-7.

53. Van Dillen LR, Sahrmann SA, Norton BJ, Caldwell CA, McDonnell MK, Bloom NJ. Movement system impairment-based categories for low back pain: stage 1 validation. J Orthop Sports Phys Ther. 2003;33(3):126-42.

54. Van Dillen LR, Sahrmann SA, Norton BJ, Caldwell CA, Fleming DA, McDonnell MK, et al. Reliability of physical examination items used for classification of patients with low back pain. Phys Ther. 1998;78(9):979-88.

55. Aefsky B, Fleet N, Myers H, Butler RJ. Reliability and Validity of a Novel Approach to Measure Hip Rotation. J Sport Rehabil. 2016;25(4):330-7.

56. Van Dillen LR, Sahrmann SA, Caldwell CA, McDonnell MK, Bloom N, Norton BJ. Trunk rotation-related impairments in people with low back pain who participated in 2 different types of leisure activities: a secondary analysis. J Orthop Sports Phys Ther. 2006;36(2):58-71.

57. Van Dillen LR, Sahrmann SA, Norton BJ, Caldwell CA, Fleming D, McDonnell MK, et al. Effect of active limb movements on symptoms in patients with low back pain. J Orthop Sports Phys Ther. 2001;31(8):402-13.

58. Biely SA, Silfies SP, Smith SS, Hicks GE. Clinical observation of standing trunk movements: what do the aberrant movement patterns tell us? J Orthop Sports Phys Ther. 2014;44(4):262-72.

59. Novy DM, Simmonds MJ, Lee CE. Physical performance tasks: what are the underlying constructs? Arch Phys Med Rehabil. 2002;83(1):44-7.

60. Simmonds MJ, Olson SL, Jones S, Hussein T, Lee CE, Novy D, et al. Psychometric characteristics and clinical usefulness of physical performance tests in patients with low back pain. Spine. 1998;23(22):2412-21.

61. McGill SM, Childs A, Liebenson C. Endurance times of low back stabilization exercises: clinical targets for testing and training from a normal database. Archives of Physical Medicine and Rehabilitation. 1999;80(8):941-4.

62. Ito T, Shirado O, Suzuki H, Takahashi M, Kaneda K, Strax TE. Lumbar trunk muscle endurance testing: An inexpensive alternative to a machine for evaluation. Archives of Physical Medicine and Rehabilitation. 1996;77:75-9.
